# Regioselective Synthesis, Structural Characterization, and Antiproliferative Activity of Novel Tetra-Substituted Phenylaminopyrazole Derivatives

**DOI:** 10.3390/molecules27185814

**Published:** 2022-09-08

**Authors:** Matteo Lusardi, Aldo Profumo, Chiara Rotolo, Erika Iervasi, Camillo Rosano, Andrea Spallarossa, Marco Ponassi

**Affiliations:** 1 Department of Pharmacy, University of Genova, Viale Benedetto XV, 3, 16132 Genova, Italy; 2 Proteomics and Mass Spectrometry Unit, IRCCS Ospedale Policlinico San Martino, Largo R. Benzi 10, 16132 Genova, Italy

**Keywords:** tetra-substituted pyrazoles, mass-spectrometry, antiproliferative activity, computational simulations

## Abstract

A small library of highly functionalized phenylaminopyrazoles, bearing different substituents at position 1, 3, and 4 of the pyrazole ring, was prepared by the one-pot condensation of active methylene reagents, phenylisothiocyanate, and substituted hydrazine (namely, methyl- and benzyl-hydrazine). The identified reaction conditions proved to be versatile and efficient. Furthermore, the evaluation of alternative stepwise protocols affected the chemo- and regio-selectivity outcome of the one-pot procedure. The chemical identities of two *N*-methyl pyrazole isomers, selected as prototypes of the whole series, were unambiguously identified by means of NMR and mass spectrometry studies. Additionally, semiempirical calculations provided a structural rationale for the different chromatographic behavior of the two isomers. The prepared tetra-substituted phenylaminopyrazoles were tested in cell-based assays on a panel of cancer and normal cell lines. The tested compounds did not show any cytotoxic effect on the selected cell lines, thus supporting their pharmaceutical potentials.

## 1. Introduction

Pyrazole represents a distinctive scaffold in medicinal chemistry [1,2,3,4,5,6,7,8,9] as pyrazole-containing compounds showed a wide spectrum of biological properties. As recently reviewed by Ebenezer and coworkers [10], pyrazoles showed anti-inflammatory activity [11,12] which was able to reduce the level of TNFα and/or the release of NO [13,14]. Furthermore, some pyrazole derivatives proved to be more potent COX-2 inhibitors than celecoxib with minimal ulcerogenic effect associated [15,16,17]. Some derivatives showed anti-inflammatory/analgesic dual activity [18,19], whereas other pyrazole compounds proved to interfere with the cannabinoid system and exert an analgesic effect [20]. Pyrazole derivatives proved also to be efficient antibacterial agents [21] able to block the proliferation of Gram-positive and Gram-negative pathogens through different mechanisms (e.g., inhibition of DNA-gyrase or DHFR enzymes) [22,23]. Additionally, a number of pyrazole derivatives showed anticancer activities [24,25] on different tumor cell lines. The molecular mechanism behind the antiproliferative activity of pyrazole compounds include the inhibition of the VEGFR-2 kinase [26], the dual blocking of cyclin-dependent kinase and histone deacetylase [27], or the targeting different signaling pathways, including ERK/MAPK and phosphatases [28]. Finally, pyrazole derivatives showed antifungal, hypoglycemic, antileishmanial, antimalaria, antituberculosis, and antioxidant properties thus supporting the pharmaceutical relevance of this heterocyclic nucleus [10].

In particular, functionalized phenylamino-substituted pyrazole derivatives proved to be effective agrochemical fungicides [29,30,31], hypoglycemic [32,33,34], and antiproliferative (Bruton’s kinase inhibitors or necroptosis-based cancer agents) [35,36,37] compounds. Interestingly, recent patents reported combination of fungicidal phenylamino pyrazoles with other compounds as novel insecticide and antibacterial agents [38,39].

**Scheme 1:**
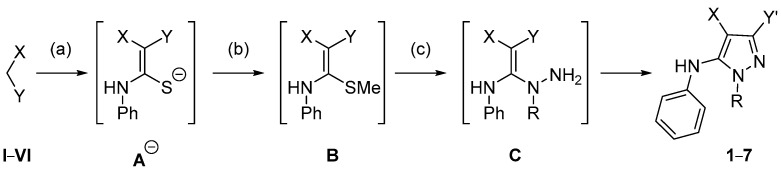
One-pot synthesis of *N-*substituted pyrazoles **1**–**7**. Reaction conditions: (**a**) DMFdry, NaH, PhNCS, rt, 1 h; (**b**) MeI, rt, 3 h; (**c**) methylhydrazine or benzylhydrazine, 100 °C, 4 h. The structures of AMRs **I**–**VI** are reported in Table 1.

According to the literature, phenylamino-substituted pyrazoles can be prepared by either the cyclization with hydrazine of a *N*,*S*-thioketal intermediate [40] or the functionalization of a thiomethyl pyrazole with a suitable aniline [41]. These protocols allowed the preparation of tri- and tetra-substituted phenylamino pyrazoles in good yields though relying on stepwise protocols. Recently, we reported the chemo-selective, one-pot synthesis of highly substituted pyrazole compounds through the condensation of an active methylene reagent (AMR), isothiocyanate, and hydrazine [42]. Among the prepared tri-substituted pyrazoles, selected derivatives showed interesting antiproliferative activity being able to selectively inhibit the growth of SkMel28 and HeLa cells without affecting the proliferation of human fibroblasts [42]. To further evaluate the versatility of the developed procedure and identify unreported synthetic strategies for the preparation of pharmaceutically attractive phenylamino pyrazoles [29,30,31,32,33,34,35,36,37,38,39], we studied the condensation of phenylisothiocyanate, AMRs, and substituted hydrazines (namely, methylhydrazine and benzylhydrazine) to afford tetra-functionalized phenylamino-substituted pyrazole derivatives **1**–**7** (Scheme 1, Table 1). Eventually, the antiproliferative/cytotoxic activities of the synthesized molecules were evaluated against a panel of eight tumor and one normal fibroblast cell lines for preliminary biological characterization.

## 2. Results and Discussion

### 2.1. Chemistry

AMRs **I**–**VI** (Table 1) were sequentially reacted with phenylisothiocyanate, methyl iodide, and the proper substituted hydrazine under the previously reported one-pot, three-step conditions (Scheme 1) [42]. Briefly, the reaction in basic condition of AMRs with the phenylisothiocyanate led to the formation of thioamide intermediates **A^−^** that were S-methylated in situ with iodomethane. The so obtained *N*,*S*-thioketal intermediates **B** were then condensed with substituted hydrazine to afford ring-opened intermediates **C** that led to the final pyrazole compounds **1**–**7** in moderate-to-good yields (Table 1).

**Table 1:** Synthesized pyrazoles and active methylene reagents employed in the synthesis.

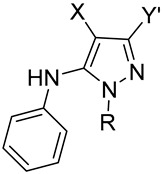						
AMR	X	Y	Cpd	Y′	R	Yield (%)
**I**	CN	CN	**1** [43]	NH_2_	Me	56
**II**	CN	COC(Me)_3_	**2**	t-Bu	Me	61
**III**	CN	COPh	**3**	Ph	Me	60
**III**	CN	COPh	**4**	Ph	CH_2_Ph	27
**IV**	COPh	COPh	**5**	Ph	Me	50
**V**	COOMe	CN	**6**	NH_2_	Me	12
**VI**	SO_2_Ph	CN	**7**	NH_2_	Me	61

Intriguingly, the tested conditions proved to be highly regio- and chemo-selective, allowing the isolation of a single *N*^1^-substituted pyrazole derivative. In particular, out of the two nucleophilic centres of methyl- or benzyl-hydrazine, the substituted nitrogen atom would selectively displace the SMe group of intermediates **B** leading to the formation of intermediate **C** (Scheme 1). As previously observed for unsubstituted hydrazine [42], the reactivity of hydrazinic NH_2_ group in intermediates **C** is selectively oriented toward X or Y group, leading to the formation of a unique *N*^1^-substituted pyrazole. Thus, when X = COOMe and Y = CN, the hydrazinic amine group attacked the nitrile group, leading to the unique isolation of the 3-aminopyrazole derivative (compound **6**, Table 1) whereas when X = CN and Y = COR, the cyclization reaction occurs on the ketone even in the presence of a relevant steric hindrance (e.g., t-Bu; compound **2**, Table 1). The chemical identities of the pyrazole derivatives were assessed by NMR analysis and the NOESY spectrum of derivative **1** showed signals at {3.40; 6.80} ppm and {3.40; 8.50} ppm, thus indicating a spatial proximity between *N*-methyl groups and phenyl and NH hydrogens, respectively.

**Scheme 2:**
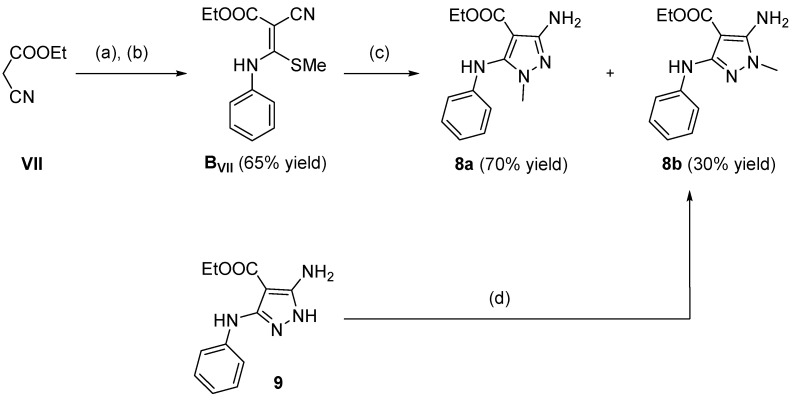
Stepwise synthesis of *N-*methylpyrazoles **8a** and **8b**. Reaction conditions: (**a**) DMF_dry_, NaH, PhNCS, rt, 2 h; (**b**) MeI, rt, 16 h; (**c**) MeNHNH_2_, 80 °C, 1.5 h; (**d**) DMF_dry_, K_2_CO_3_, MeI, rt, 16 h.

To further investigate the regioselectivity of the reaction, the synthesis of pyrazole **8** (closely related to derivative **6**) was carried out in a stepwise fashion (Scheme 2). Thus, ethyl cyanoacetate **VII** was condensed with phenyl isothiocyanate in the presence of NaH and then S-methylated. The so-obtained *N*,*S*-thioketal **B_VII_** (yield: 65%) is a push–pull alkene bearing two electron withdrawing groups at one end of the double bond (i.e., COOEt and CN) and two electron donating substituents at the other end (i.e., NHPh and SMe). This arrangement promotes the π delocalization from the electron-donating groups (‘push’ terminus) to the electron-withdrawing groups (‘pull’ terminus) thus lowering the energetic barrier to C=C rotation [44,45,46,47,48,49] and enhancing the reactivity of push–pull alkenes with nucleophilic and electrophilic species. For these reasons, push–pull alkenes are versatile synthons used for the preparation of various chemical heterocyclic derivatives [49,50,51]. Differently from the one-pot procedure, the reaction between **B_VI_** and methylhydrazine in solvent free conditions led to the formation of a mixture of the two *N-*methyl pyrazole isomers **8a** and **8b** that were isolated in 30% and 70% quantitative yield, respectively. The two pyrazole compounds were separated by column chromatography and their chemical identity was unambiguously identified by NMR and mass spectrometry analyses.

Additionally, the methylation of pyrazole **9** (obtained as previously reported [42]) in the presence of K_2_CO_3_ has been evaluated as an alternative procedure to afford compounds **8** (Scheme 2). Surprisingly, in the adopted conditions only compound **8b** has been isolated, thus highlighting the effect of the reaction conditions on the regioselective outcome of the procedure.

### 2.2. NMR and Mass Spectrometry Analyses of Derivatives **8a** and **8b**

**Figure 1:**
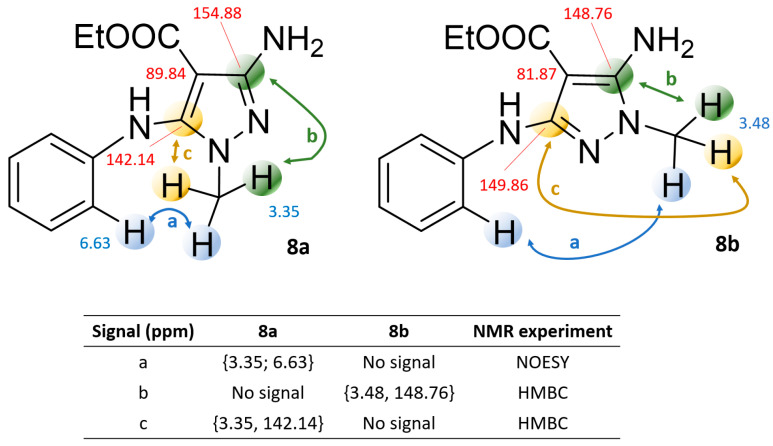
Relevant NMR couplings observed in NOESY and HMBC spectra of **8** regio-isomers. The interacting nuclei are differently colored, and the different interactions are indicated by double-headed arrows. The relevant chemical shift values are reported in red (carbon atom) and blue (proton atom).

The comparative analysis of the 2D-NMR spectra collected for **8a** and **8b** allowed the unambiguous assignment of the chemical structure of two pyrazole regioisomers. In particular, derivative **8a** displayed a signal at {3.35; 6.63} ppm in the NOESY spectrum related to the interaction between the spatially closed *N*-methyl and phenyl hydrogens (signal a, Figure 1). This signal is absent in the spectrum of **8b**, given the different relative position of the two groups. Furthermore, the interaction between the *N*-methyl hydrogens and the pyrazole carbon through a J^3^_C-H_ coupling constant observed in the HMBC spectra further supported the identification of the two isomers (signals b and c, Figure 1). In particular, the peak observed at {3.35, 142.14} ppm refers to the heteronuclear coupling between the *N-*methyl hydrogens and the pyrazole carbon atom substituted with the *N-*phenyl group (i.e., compound **8a**) whereas the signal at {3.48, 148.76} ppm is related to the coupling between the methyl hydrogens and the pyrazole atom bearing the amine group (i.e., compound **8b**).

**Scheme 3:**
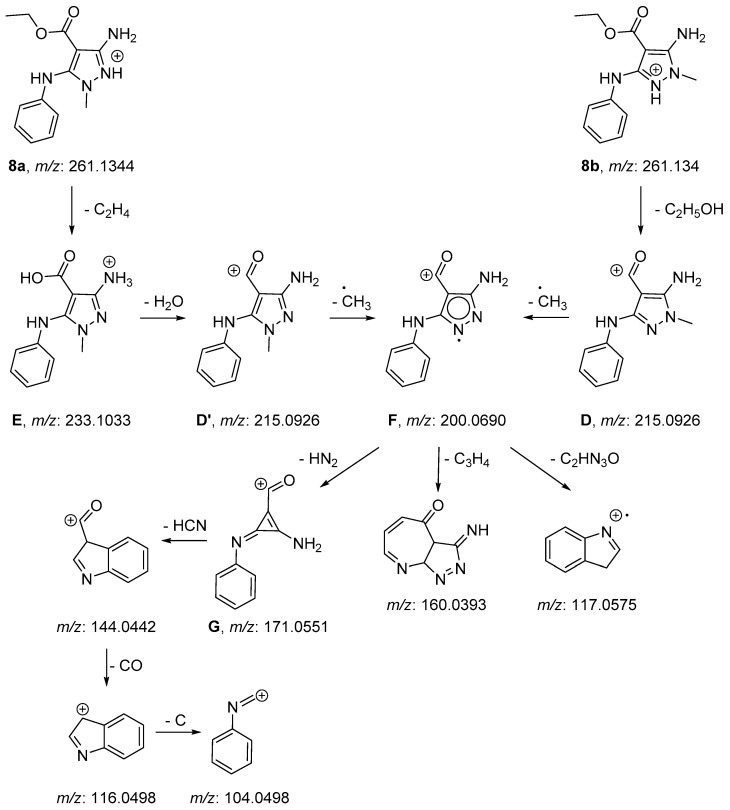
Proposed fragmentation of isomers **8a** and **8b**.

In order to confirm the different structure of the two isomers **8a** and **8b** (Scheme 2), a complete flow injection analysis (FIA) mass spectrometry characterization was carried out. The protonated molecule ions [M + H]^+^ of both analytes were identified in the full scan spectra at *m*/*z* 261.1344 (Figure S32, Supplementary Material); then, each molecule ion was subjected to collision-induced dissociation (CID) separately to produce fragment ions. The main fragment generated at the collision energy of 60 eV was *m*/*z* 215.0926 for both species (**D** and **D′**, Scheme 3). Interestingly, the two isomers showed a different behavior in the MS^2^ spectrum as **8a** (and not its isomer **8b**) led to the formation of the transient intermediate species at *m*/*z* 233.1033 (Figure S33, Supplementary Material). This observation highlighted a different susceptibility to the collision energy between the two compounds. In particular, the fragmentation of **8b** C(O)-O ester bond would directly afford the pyrazole acyl ion **D** (Scheme 3) and ethanol. Conversely, **8a** would form **D′** by a two-step fragmentation pattern that involved the initial cleavage of the ethyl group (and the consequent formation of the carboxylic acid intermediate **E**; Scheme 3) followed by the elimination of a water molecule. Q-Exactive plus does not allow us to run canonical MS^n^ experiments. However, we can set up a ‘non-specific’ in source fragmentation step able to ascertain, in the full scan analysis, the characteristic fragment ion identified during the first MS^2^ experiments. Thus, we selected *m*/*z* 215.0926 ion as the progenitor fragment and a MS^3^ experiment was carried out. This protocol has been repeated for both isomers and for all of the successive MS^n^ analyses. This new fragmentation generates a main product ion at *m*/*z* 200.0690 (fragment **F**, Scheme 3), compatible with the loss of the pyrazole methyl group. It is worth noting that **8a** and **8b** led to the formation of this common fragment that yielded the same ion panel, comprising *m*/*z* 117.0575, *m*/*z* 160.0393 and *m*/*z* 171.0551. The latter fragment would be probably due to the rearrangement of pyrazole ring to obtain a cyclopropenyl ring after the loss of two nitrogen atoms (fragment **G**, Scheme 3) [52]. Further fragmentation of ion **G** led to the formation of fragments *m*/*z* 144.0442, *m*/*z* 116.0497, and *m*/*z* 104.0498 possibly as the sequential loss of HCN, CO, and a carbon atom (Scheme 3).

**Scheme 4:**
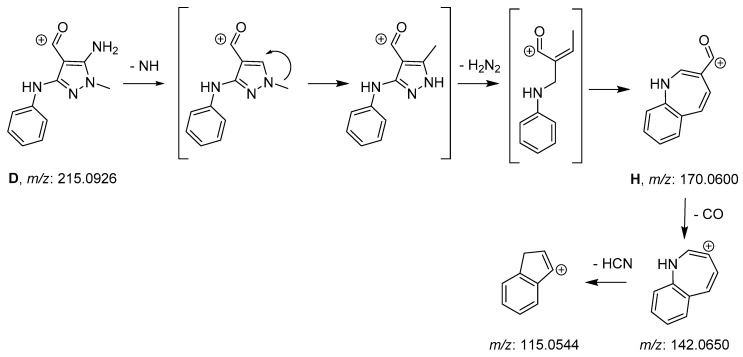
Proposed fragmentation of ion **D**.

Moreover, the MS^3^ analysis of the *m*/*z* 215.0926 ions evidenced that **8b**-derived molecules (namely ion **D**, Scheme 3) can undergo to an alternative fragmentation, generating an ion fragment at *m*/*z* 170.0600 which was not observed in the case of the **8a** (Figure S34, Supplementary Material). This further difference in the behavior between the MS^2^ products of the two isomers can be explained by the partial elimination of the amino-group from the pyrazole ring, followed by the N-C transposition of the methyl group (Scheme 4). The C-methylated intermediate was not identified in our study, but it has been reported in the literature for other pyrazole compounds structurally related to derivatives **8a** and **8b** [52]. The elimination of N_2_H_2_ would generate an unstable intermediate that cyclizes to form the benzoazepine fragment **H** (Scheme 4). On the basis of the collected data, we can speculate that the rearrangement of molecule **D** (*m*/*z* 215.0926) to afford fragment **H** (Scheme 4) can only occur if the *N-*methyl and amino groups are in close proximity, thus confirming the chemical identity of **8b**. The same transposition would be disadvantaged when these two groups are not in adjacent positions, as with compound **8a**.

**Figure 2:**
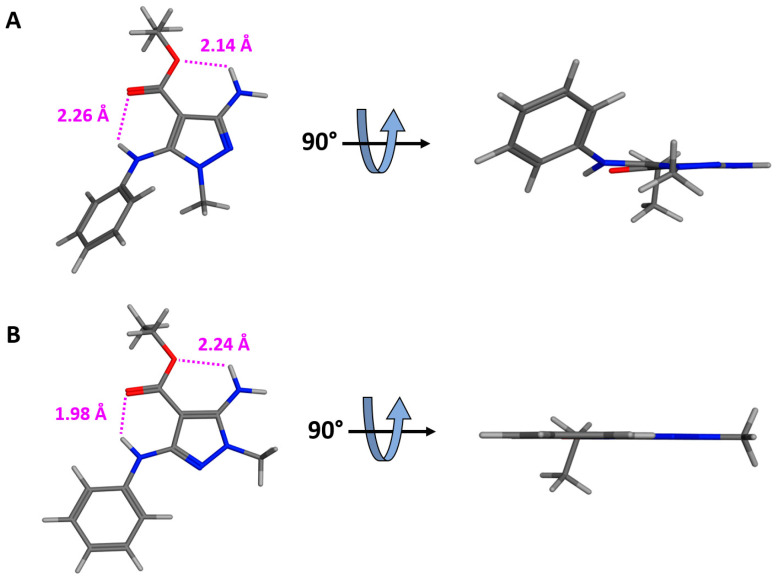
Low energy conformers of **8a** (panel (**A**)) and **8b** (panel (**B**)). The intramolecular hydrogen bonds are represented as purple dotted lines. The O-H distances are also reported.

The different chemical identity of compounds **8a** and **8b** would also affect the retention times of the two isomers (t(r)**_8a_** = 13.70 min; t(r)**_8b_** = 20.03 min) during the reverse phase (RP) HPLC analysis. In fact, in compound **8a** the pyrazole *N-*methyl group would prevent the adjacent phenyl ring to lay on the same plane of the pyrazole nucleus thus reducing the compound’s affinity for the RP stationary phase. Conversely, in derivative **8b** the pyrazole *N-*methyl group would not influence the geometry of the *N-*phenyl ring that therefore would be co-planar to the heterocyclic portion. To further support this observation, semiempirical computational simulations (MOE software) were carried out on the two isomers (Figure 2). In the minimum energy conformers of **8a** and **8b**, the phenyl and pyrazole rings would assume different reciprocal orientations being co-planar in compound **8b** and laying on two different planes in derivative **8a**. The methyl-induced distortion of the phenyl ring would also affect the intramolecular hydrogen-bonding in the two conformers, as indicated by the different C=O···HN distances and geometries in the two conformers (Figure 2).

### 2.3. Antiproliferative Activity

To evaluate their effect on cell proliferation, phenylamino-pyrazole derivatives **1**–**8** were preliminarily tested using the MTT assay. A panel of eight tumor cell lines (namely, breast cancer: MCF7, MDA-MB231, SK-Br3; melanoma: SKMEL-28; ovarian cancer: SKOV-3; liver cancer: Hep-G2; cervical cancer: HeLa; lung cancer: A549) and one normal human fibroblasts cell line (GM-6114) was considered. The mean growth percentage values were determined at the fixed concentration of 10 μM. Cisplatin was used as reference drug.

The results showed in Table 2 demonstrated that all tested compounds did not exhibit significant antiproliferative activity (grow inhibition percentage values higher than 68.36%) against the considered cancer cell lines. Moreover, all compounds proved to be non-cytotoxic against the human fibroblasts GM-6114 cell line at the concentration of 10 μM (mean growth percentage range: 84.57–109.73%). Interestingly, derivatives **1**–**8** showed similar mean growth percentage values with previously synthesized *N*-unsubstituted analogs [42], thus highlighting that *N*-alkylation of the pyrazole nucleus marginally affected the antiproliferative/cytotoxic properties of the series.

## 3. Materials and Methods

### 3.1. Chemistry

Commercially available active methylene reagents, phenyl isothiocyanate, substituted hydrazine and reagents (55% sodium hydride dispersion in mineral oil, iodomethane) were purchased by Alfa-Aesar and Sigma-Aldrich. DMF was reagent grade and was dried on molecular sieves (5 Å 1/16″ inch pellets). Unless otherwise stated, all commercial reagents were used without further purification. Organic solutions were dried over anhydrous sodium sulphate. A thin layer chromatography (TLC) system for routine monitoring the course of reactions and confirming the purity of analytical samples employed aluminium-backed silica gel plates (Merck DC-Alufolien Kieselgel 60 F254). DCM or DCM/2% methanol were used as a developing solvent and detection of spots was made by UV light and/or by iodine vapors. Melting points were determined on a Fisher-Johns apparatus and are uncorrected. ^1^H NMR and ^13^C NMR spectra were recorded on a Varian Gemini (Palo Alto, CA, USA) or JEOL JNM-ECZR (Tokyo, Japan) instrument; chemical shifts were reported in δ (ppm) units relative to the internal reference tetramethylsilane, and the splitting patterns were described as follows: s (singlet), bs (broad singlet), d (doublet), t (triplet), q (quartet), and m (multiplet). The first order values reported for coupling constants J were given in Hz. Elemental analyses were performed by an EA1110 Analyzer, Fison Instruments (Milan, Italy).

### 3.2. General Synthetic Procedure for the Preparation of Pyrazoles **1**–**7**

To a dry DMF (10 mL) solution of the proper active methylene reagent (10 mmol), 55% sodium hydride dispersion in mineral oil (0.44 g, 10 mmol) was added under stirring at rt. After 45 min phenylisothiocyanate (1221 μL, 10 mmol) was added in a single portion. The reaction mixture was stirred for 1 h at rt, then iodomethane (629 μL, 10 mmol) was added. After 3 h, the reaction mixture was treated with the proper substituted hydrazine (25 mmol) at rt and was heated at 95–100 °C for 4 h. The reaction mixture was diluted with water (150 mL) and extracted with dichloromethane (3 × 30 mL). The combined extracts were washed with water (5 × 30 mL), dried with anhydrous Na_2_SO_4_, and filtered. Evaporating in vacuo gave a residue that was purified by crystallization from the suitable solvent or solvent mixture.

*3-amino-1-methyl-5-(pheylamino)-1H-pyrazole-4-carbonitrile (***1***).* White solid. Mp 210–212 °C (DCM-MeOH); Yield: 56%. ^1^H NMR (400 MHz, DMSO-*d*_6_): δ 3.40 (s, 3H, CH_3_N); 5.42 (bs, 2H, NH_2_); 6.77–6.82 (m, 2H, arom. H); 6.83–6.89 (m, 1H, arom. H); 7.20–7.26 (m, 2H, arom. H); 8.50 (bs, 1H, NH phenyl). ^13^C NMR (101 MHz, DMSO-*d*_6_) δ 34.80, 69.74, 114.44, 115.69, 120.38, 129.25, 142.52, 144.40, 155.68. HRMS (ESI/APCI) *m*/*z* [M + H]^+^ for C_11_H_11_N_5_ calcd 214.1087, found 214.1089. Calcd for C_11_H_11_N_5_: C = 61.96; H = 5.20; N = 32.84. Found: C = 61.60; H = 5.50; N = 32.96.

*3-(tert-butyl)-1-methyl-5-(pheylamino)-1H-pyrazole-4-carbonitrile (***2***).* White solid. Mp 157–159 °C (DCM); Yield: 61%. ^1^H NMR (400 MHz, DMSO-*d*_6_): δ 1.33 (s, 9H, t-Bu); 3.59 (s, 3H, CH_3_N); 6.80–6.90 and 7.23–7.27 (m, 5H, arom. H); 8.62 (bs, 1H, NH, exchangeable). ^13^C NMR (101 MHz, DMSO-*d*_6_) δ 160.46; 146.95; 142.23; 129.25; 120.66; 115.87; 114.80; 79.16; 35.44; 33.17; 28.88. HRMS (ESI/APCI) *m*/*z* [M + H]^+^ for C_15_H_18_N_4_ calcd 255.1604, found 255.1606. Calcd for C_15_H_18_N_4_: C = 70.84; H = 7.13; N = 22.03. Found: C = 70.96; H = 7.45; N = 22.06.

*1-methyl-3-phenyl-5-(phenylamino)-1H-pyrazole-4-carbonitrile (***3***).* Yellow solid. Mp 229–231 °C (Ether-DCM); Yield: 60%. ^1^H NMR (400 MHz, DMSO-*d*_6_): δ 3.74 (s, 3H, CH_3_N); 6.92–6.96 (m, 3H, arom. H); 7.26–7.32 (m, 2H, arom. H); 7.41–7.53 (m, 3H, arom. H); 7.83–7.87 (m, 2H, arom. H); 8.86 (bs, 1H, NH, exchangeable). ^13^C NMR (101 MHz, DMSO-*d*_6_) δ 35.36, 72.30, 115.81, 117.88, 122.48, 127.52, 128.87, 129.19, 129.47, 131.06, 140.90, 146.96, 147.94. HRMS (ESI/APCI) *m*/*z* [M + H]^+^ for C_17_H_14_N_4_ calcd 275.1291, found 275.1293. Calcd for C_17_H_14_N_4_: C = 74.43; H = 5.14; N = 20.42. Found: C = 74.38; H = 5.47; N = 20.38.

*1-benzyl-3-phenyl-5-(phenylamino)-1H-pyrazole-4-carbonitrile (***4***).* White solid. Mp 167–169 °C (Ether-DCM); Yield: 27%. ^1^H NMR (400 MHz, DMSO-*d*_6_): δ 5.26 (s, 2H, CH_2_Ph); 6.82–6.86 and 7.06–7.08 and 7.20–7.34 and 7.52–7.59 (m, 15H, arom. H); 8.98 (bs, 1H, NH, exchangeable). ^13^C NMR (101 MHz, DMSO-*d*_6_) δ 152.23; 148.43; 141.89; 136.49; 130.44; 129.26; 128.98; 128.69; 128.66; 127.66; 126.81; 126.58; 120.02; 116.49; 114.12; 80.51; 52.86. HRMS (ESI/APCI) *m*/*z* [M + H]^+^ for C_23_H_18_N_4_ calcd 351.1604, found 351.1603. Calcd for C_23_H_18_N_4_: C = 78.83; H = 5.18; N = 15.99. Found: C = 78.62; H = 4.94; N =1 5.79.

*(1-methyl-3-phenyl-5-(phenylamino)-1H-pyrazol-4-yl)(phenyl)methanone (***5***).* White solid. Mp 140–142 °C (DCM-MeOH); Yield: 50%. ^1^H NMR (400 MHz, DMSO-*d*_6_): δ 3.66 (s, 3H, CH_3_N); 6.90–7.02 and 7.15–7.34 and 7.68–7.70 (m, 15H, arom. H); 9.22 (bs, 1H, NH, exchangeable). ^13^C NMR (101 MHz, DMSO-*d*_6_) δ 191.81; 152.84; 145.60; 141.10; 138.70; 130.49; 130.20; 128.99; 128.89; 128.00; 127.98; 127.25; 120.23; 116.82; 106.23; 37.16. HRMS (ESI/APCI) *m*/*z* [M + H]^+^ for C_23_H_19_N_3_O calcd 354.1601, found 354.1601. Calcd for C_23_H_19_N_3_O: C = 78.16; H = 5.42; N = 11.89. Found: C = 78.06; H = 5.76; N = 11.86.

*Methyl 3-amino-1-methyl-5-(phenylamino)-1H-pyrazole-4-carboxylate (***6***).* White solid. Mp 151–153 °C (DCM-MeOH); Yield: 12%. ^1^H NMR (400 MHz, DMSO-*d*_6_): δ 3.32 (s, 3H, NCH_3_); 3.56 (s, 3H, CH_3_O); 5.32 (bs, 2H, NH_2_, exchangeable); 6.62–6.68 (m, 2H, arom. H); 6.76–6.72 (m, 1H, arom. H); 7.15–7.21 (m, 2H, arom. H); 7.91 (bs, 1H, NH, exchangeable). ^13^C NMR (101 MHz, DMSO-*d*_6_) δ 34.86, 50.28, 89.62, 115.02, 119.54, 129.08, 142.21, 144.40, 154.97, 163.72. HRMS (ESI/APCI) *m*/*z* [M + H]^+^ for C_12_H_14_N_4_O_2_ calcd 247.1190, found 247.1190. Calcd for C_12_H_14_N_4_O_2_: C = 58.53; H = 5.73; N = 22.75. Found: C = 58.84; H = 5.42; N = 22.43.

*1-methyl-N^5^-phenyl-4-(phenylsulfonyl)-1H-pyrazole-3,5-diamine (***7***).* White solid. Mp 203–205 °C (DCM); Yield: 60%. ^1^H NMR (400 MHz, DMSO-*d*_6_): δ 3.27 (s, 3H, CH_3_N); 5.38 (bs, 2H, NH_2_, exchangeable); 6.45–6.47 and 6.75–6.78 and 7.07–7.11 and 7.37–7.40 and 7.51–7.55 and 7.69–7.72 (m, 10H, arom. H); 7.95 (bs, 1H, NH, exchangeable). ^13^C NMR (101 MHz, DMSO-*d*_6_): δ 152.19; 144.13; 143.21; 140.29; 132.69; 129.12; 128.90; 125.82; 119.51; 114.52; 98.12; 34.82. HRMS (ESI/APCI) *m*/*z* [M + H]^+^ for for C_16_H_16_N_4_O_2_S calcd 329.1067, found 329.1064. Calcd for C_16_H_16_N_4_O_2_S: C = 58.52; H = 4.91; N = 17.06; S = 9.76. Found: C = 58.64; H = 4.92; N = 17.13; S = 9.37.

### 3.3. Synthesis of Ethyl 2-Cyano-3-(methylthio)-3-(phenylamino)acrylate (B_VI_)

To a dry DMF (15 mL) solution of ethyl cyanoacetate (1085 μL, 10 mmol) cooled at 0 °C, 55% sodium hydride dispersion in mineral oil (0.44 g, 10 mmol) was added in a single portion. The reaction mixture is stirred for 10 min at 0 °C and phenylisothiocyanate (1221 μL, 10 mmol) was added and stirring was prolonged for 2h. Methyl iodide (629 μL, 10 mmol) was added and the mixture was stirred at rt for 16h. The reaction mixture was diluted with water (50 mL) and a yellow solid precipitated. The crude material was collected by filtration, dried, and used without further purification.

Mp 83–85 °C (water) (litt [53]: 82 °C); Yield: 65%. ^1^H NMR (200 MHz, CDCl_3_): δ 1.34 (t, 3H, J = 7.2 Hz, CH_3_); 2.23 (s, 3H, SCH_3_); 4.27 (q, 2H, J = 7.2 Hz, CH_2_O); 7.21–7.49 (m, 5H, arom. H); 11.51 (bs, 1H, NH, exchangeable). Calcd for C_13_H_14_N_2_O_2_S: C = 59.52; H = 5.38; N = 10.68; S = 12.22. Found: C = 59.82; H = 5.42; N = 10.60; S = 11.37.

### 3.4. Synthesis of Compounds **8a** and **8b**

A mixture of **B_VI_** (2.67 g, 10 mmol) and methylhydrazine (590 μL, 11 mmol) was heated in a sealed tube at 80 °C for 1.5 h. The mixture was cooled at rt and water (10 mL) was added. A white solid precipitated and was collected by filtration. TLC analysis (eluent DCM/2% methanol mixture) revealed two spots with Rf values of 0.11 (compound **8a**) and 0.30 (compound **8b**). The solid was dissolved in DCM and the two compounds were separated by column chromatography (silica gel, eluent: DCM-DCM/20% MeOH).

*Ethyl 3-amino-1-methyl-5-(phenylamino)-1H-pyrazole-4-carboxylate (***8a***).* White solid. Mp 138–140 °C (EtOH); Yield: 70%. ^1^H NMR (400 MHz, DMSO-*d*_6_): δ 0.94 (t, 3H, J = 7.1 Hz, CH_3_-C); 3.35 (s, 3H, CH_3_N); 3.96 (q, 2H, J = 7.1 Hz, CH_2_O); 4.71 (bs, 2H, NH_2_, exchangeable); 6.61–6.69 (m, 2H, arom. H); 6.74–6.85 (m, 1H, arom. H); 7.12–7.22 (m, 2H, arom. H); 7.93 (bs, 1H, NH, exchangeable). ^13^C NMR (101 MHz, DMSO-*d*_6_) δ 13.91, 34.73, 58.71, 89.84, 114.91, 119.42, 129.02, 142.14, 144.62, 154.88, 163.33. HRMS (ESI/APCI) *m*/*z* [M + H]^+^ for C_13_H_16_N_4_O_2_ calcd 261.1346, found 261.1344. Calcd for C_13_H_16_N_4_O_2_: C = 59.99; H = 6.20; N = 21.52. Found: C = 60.20; H = 6.10; N = 21.86.

*Ethyl 5-amino-1-methyl-3-(phenylamino)-1H-pyrazole-4-carboxylate (***8b***).* White solid. Mp 144–145 °C (EtOH); Yield: 30%. ^1^H NMR (400 MHz, DMSO-*d*_6_): δ 1.30 (t, 3H, J = 7.1 Hz, CH_3_-C); 3.48 (s, 3H, CH_3_N); 4.25 (q, 2H, J = 7.1 Hz, CH_2_O); 6.23 (bs, 2H, NH_2_, exchangeable); 6.78–6.87 (m, 1H, arom. H); 7.18–7.28 (m, 2H, arom. H); 7.50–7.55 (m, 2H, arom. H); 8.05 (bs, 1H, NH, exchangeable). ^13^C NMR (101 MHz, DMSO-*d*_6_) δ 14.56, 33.95, 58.91, 81.87, 116.27, 119.59, 128.81, 141.36, 148.76, 149.86, 163.95. HRMS (ESI/APCI) *m*/*z* [M + H]^+^ for C_13_H_16_N_4_O_2_ calcd 261.1346, found 261.1344. Calcd for C_13_H_16_N_4_O_2_: C = 59.99; H = 6.20; N = 21.52. Found: C = 60.18; H = 6.15; N = 21.32.

### 3.5. Synthesis of Compound **8b** via Pyrazole Methylation

A dry DMF solution (5 mL) of pyrazole **9** [26] (377 mg, 1.5 mmol) and anhydrous K_2_CO_3_ (251 mg, 1.8 mmol) was stirred at rt for 10 min. Methyl iodide (94 μL, 1.5 mmol) was added and the suspension was stirred at rt for 16h. The sequential addition of water (10 mL) and solid ammonium chloride (pH = 7) led to the isolation of a white solid. Purification by crystallization from DCM/EtOH mixture afforded 118 mg (30% yield) of compound **8b**.

### 3.6. Mass Spectrometry Analysis

#### 3.6.1. LC-HRMS

The two isomers were analyzed by high pressure liquid chromatography conducted using a Vanquish (Thermo Fisher Scientific, San Jose, CA, USA) UHPLC system composed of binary pump, autosampler, and column oven. In details, 10 μL of a 1:1 mixture of the two isomers (concentration 1 μM each) was injected onto a Simmetry 300 C18 column (150 × 1 mm, 3.5 μm particle size) (Waters) maintained at 25 °C. The eluents were 0.1% formic acid (eluent A) and acetonitrile (eluent B). Flow rate was 100 μL/min. The mobile phase was a binary linear gradient in the following sequence: isocratic 20% B for five min, a linear gradient over the course of 60 min to 100% B, maintained at 100% B for 10 min and finally a linear gradient to 20% B in one min. The re-equilibration time in 20% B was 15 min. After HPLC separation, the eluent was directly sent to a Q Exactive Plus Orbitrap mass spectrometer (ThermoScientific, San Jose, CA, USA) equipped with a heated electrospray ionization source (HESI-II). Before analyses, the mass spectrometer was externally calibrated with the positive ion calibration solution (Thermo Fisher Scientific). Positive full-scan mass spectra were recorded in the mass range *m*/*z* 100–400, at resolution 35,000. The following operating parameters were applied: sheath and auxiliary gas flow rate were 35 and 10 respectively; spray voltage 3.5 kV; S-lens RF level 100; capillary temperature 250 °C. The autogain control (AGC) was optimized at 10^6^ with a maximum injection time (maxIT) of 250 ms. Software used for operating the UHPLC/HR-MS was Xcalibur (version 4.1). The full scan data were processed and the identity of the isomers was confirmed by comparing the high-resolution experimental data with their theoretical molecular weight.

#### 3.6.2. FIA MS/MS

For MS/MS spectra collection, each sample was dissolved in DMSO (final concentration: 10 mM) and, after further dilution in acetonitrile (final concentration 100 nM), it was analyzed by flow injection mass spectrometry (FIA-MS). Briefly, five microliters of sample were injected into an eluent flow containing 0.1% formic acid in acetonitrile, generated by a Vanquish UHPLC system (Thermo Fisher Scientific, San Jose, CA, USA). The flow rate was 100 µL/min. The eluent was directly sent to a Q Exactive™ Plus Hybrid Quadrupole-Orbitrap™ Mass Spectrometer (Thermo Fisher Scientific, San Jose, CA, USA) equipped with a heated electrospray ion source (HESI-II). Prior to each series of acquisitions, the mass spectrometer was externally calibrated with Positive Ion Calibration Solution (Thermo Fisher Scientific, San Jose, CA, USA). The same MS operating parameters as LC-MS analysis were applied. Full scan data were processed with Xcalibur version 4.1 (Thermo Fisher Scientific, San Jose, CA, USA). High-resolution mass spectra, ranging from 100 to 600 *m*/*z*, were acquired in positive ion mode.

#### 3.6.3. Biology

MTT assay was accomplished on a group of eight tumor cell lines: SKOV-3 (ovarian adenocarcinoma, ATCC, Manassas, VA, USA); MCF-7 (breast adenocarcinoma, Biologic Bank and Cell Factory, IRCCS Policlinico San Martino, Genoa, Italy); Hep-G2 (hepatocellular carcinoma, ATCC, Manassas, VA, USA); SK-Mel28 (skin melanoma, Biologic Bank and Cell Factory, IRCCS Policlinico San Martino, Genoa, Italy); MDA-MB231 (breast adenocarcinoma, Biologic Bank and Cell Factory, IRCCS Policlinico San Martino, Genoa, Italy); HeLa (cervical adenocarcinoma, Biologic Bank and Cell Factory, IRCCS Policlinico San Martino, Genoa, Italy); SK-BR3 (breast adenocarcinoma, Biologic Bank and Cell Factory, IRCCS Policlinico San Martino, Genoa, Italy); A549 (lung carcinoma, Biologic Bank and Cell Factory, IRCCS Policlinico San Martino, Genoa, Italy) and one normal cell line: Gm-6114 (embryonic human fibroblast, ATCC, Manassas, VA, USA). All cell lines were grown in DMEM (with 10% FBS, 2 mM Glutamine and 1% penstrep. All reagents were purchased from EuroClone (Milan, Italy), incubated at 37 °C with 5% CO_2_ and humidified environment. Briefly, the nine cell lines were plated in 96 well plates at a proper cell density to achieve about 85% of confluence at the end of the protocol. The next day, the chemical compounds were dissolved in DMSO at a concentration of 10 mM. This stock solution was diluted using the complete growth medium and added to the wells to obtain the final working concentration of 10 μM. After an incubation of 48 h, we add 30 μL of MTT (3-(4,5-dimethyl-2-thiazolyl)-2,5-diphenyl-2*H*-tetrazolium bromide) diluted at 2 mg/mL with 1× PBS. After 4 h of incubation, the surnatant was eliminated and 100 μL/well of DMSO were used to solubilize the formazan precipitate. Then, subsequent to a 20 min incubation, the OD were measured at 570 nm using a plate reader. The results are expressed as a percentage of the control samples (100%) in which the cell lines were incubated with the same amount of solvent but without any chemical compounds. The assay was repeated three times. In each set, every single compound was tested six times. Means and standard deviations were calculated.

### 3.7. Computational Calculations

The chemical structures of compounds **8a** and **8b** were drawn with MOE2009.10 (builder module) and energy minimization was carried out according to AM1, as implemented in MOE software version 2009.10. The calculations were run on a Linux PC (Intel^®^ processor Core™ i7-2600 CPU@3.40 GHz).

## 4. Conclusions

A series of novel tetrasubstituted phenylamino pyrazoles has been prepared by the one-pot condensation of AMR, phenyl isothiocyanate, and substituted hydrazines. The adopted synthetic procedure proved to be versatile and efficient as demonstrated by the various properties of the AMR groups and the different steric hindrance of the substituted hydrazine compounds. Additionally, the developed protocol proved to be regio- and chemo-selective, allowing the isolation of compounds **1**–**7** as single *N-*substituted pyrazole isomer. The regioselectivity of the one-pot procedure was further studied by adopting a stepwise protocol. The condensation of the *N*,*S*-thioketal **B_VI_** with methylhydrazine led to the formation of the two *N-*methyl pyrazole isomers **8a** and **8b** which were separated and fully characterized by NMR and mass spectrometry analyses. Conversely, the methylation of *N-*unsubstituted pyrazole **9** allowed the isolation of a single *N-*methyl derivative (namely, compound **8b**). In preliminary cell-based assays, the prepared compounds proved to be poorly cytotoxic against both a panel of mutated cell lines and normal human fibroblasts. Overall, the results of the current study further extend the applicability of the previously developed one-pot procedure and provided alternative synthetic routes for the regioselective synthesis of pharmaceutically attractive phenylaminopyrazole compounds.

## Figures and Tables

**Table 2:** Antiproliferative activity of pyrazoles **1**–**8** ^a^.

Cpd	Mean Growth Percentage ± SD								
	MCF7	MDA-MB231	Sk-Br3	SKMEL28	SKOV3	Hep-G2	HeLa	A549	GM-6114
**1**	108.43 ± 7.38	113.77 ± 4.39	92.72 ± 2.94	91.62 ± 3.38	107.78 ± 3.35	105.55 ± 7.40	97.39 ± 10.47	111.66 ± 3.77	89.08 ± 11.38
**2**	107.37 ± 10.99	112.16 ± 6.85	85.66 ± 3.28	92.87 ± 4.06	107.12 ± 5.64	94.15 ± 3.81	96.42 ± 7.05	113.10 ± 5.64	90.65 ± 3.32
**3**	118.46 ± 6.19	115.98 ± 2.45	83.05 ± 2.41	92.97 ± 3.68	115.25 ± 3.37	112.20 ± 7.47	110.40 ± 2.49	115.25 ± 5.96	109.73 ± 8.12
**4**	92.21 ± 5.41	89.69 ± 2.90	84.72 ± 4.38	68.36 ± 3.13	80.45 ± 3.38	105.51 ± 8.80	73.77 ± 6.86	71.00 ± 3.40	83.00 ± 6.51
**5**	111.88 ± 5.19	111.89 ± 2.95	101.95 ± 3.18	119.57 ± 1.57	105.35 ± 6.08	110.03 ± 2.11	115.87 ± 9.17	112.22 ± 4.10	102.18 ± 8.31
**6**	105.46 ± 5.68	109.32 ± 6.43	81.74 ± 4.92	105.04 ± 2.76	109.12 ± 5.64	102.04 ± 4.42	92.32 ± 2.62	121.90 ± 5.92	84.57 ± 4.03
**7**	104.20 ± 3.10	110.14 ± 4.88	89.26 ± 3.54	109.99 ± 3.30	106.54 ± 4.47	107.16 ± 12.07	85.42 ± 6.98	115.77 ± 3.02	87.45 ± 7.73
**8a**	98.06 ± 3.31	87.02 ± 5.76	94.76 ± 4.00	82.21 ± 4.00	106.46 ± 6.18	101.20 ± 9.16	103.26 ± 9.57	99.70 ± 9.20	107.65 ± 3.92
**8b**	100.13 ± 6.20	89.99 ± 5.16	100.28 ± 4.10	81.21 ± 4.31	102.97 ± 4.37	98.37 ± 8.17	110.61 ± 7.72	86.65 ± 9.83	118.99 ± 9.70
**cisplatin**	72.74 ± 5.48	86.07 ± 7.04	70.59 ± 3.83	44.40 ± 2.53	36.83 ± 4.35	38.07 ± 2.22	29.33 ± 2.23	59.09 ± 6.03	39.52 ± 2.74

^a^ Data mean values for three separate experiments.

## Data Availability

Not applicable.

## Supplementary Materials

The following supporting information can be downloaded at: https://www.mdpi.com/article/10.3390/molecules27185814/s1, Figure S1: ^1^H-NMR (400 MHz, *d*_6_-DMSO) spectrum of compound **1**; Figure S2: ^13^C-NMR (101 MHz, *d*_6_-DMSO) spectrum of compound **1**; Figure S3: 2D NOESY (*d*_6_-DMSO) spectrum of compound 1; Figure S4: 2D HMBC (*d*_6_-DMSO) spectrum of compound **1**; Figure S5: Fullscan analysis of compound **1**; Figure S6: ^1^H-NMR (400 MHz, *d*_6_-DMSO) spectrum of compound **2**; Figure S7: ^13^C-NMR (101 MHz, *d*_6_-DMSO) spectrum of compound **2**; Figure S8: Fullscan analysis of compound 2; Figure S9: 1H-NMR (400 MHz, *d*_6_-DMSO) spectrum of compound 3; Figure S10: 13C-NMR (101 MHz, *d*_6_-DMSO) spectrum of compound 3; Figure S11: Fullscan analysis of compound **3**; Figure S12: ^1^H-NMR (400 MHz, *d*_6_-DMSO) spectrum of compound **4**; Figure S13: ^13^C-NMR (101 MHz, *d*_6_-DMSO) spectrum of compound **4**; Figure S14: Fullscan analysis of compound **4**; Figure S15: ^1^H-NMR (400 MHz, *d*_6_-DMSO) spectrum of compound **5**; Figure S16: ^13^C-NMR (101 MHz, *d*_6_-DMSO) spectrum of compound **5**; Figure S17: Fullscan analysis of compound **5**; Figure S18: ^1^H-NMR (400 MHz, *d*_6_-DMSO) spectrum of compound **6**; Figure S19: ^13^C-NMR (101 MHz, *d*_6_-DMSO) spectrum of compound **6**; Figure S20: Fullscan analysis of compound **6**; Figure S21: ^1^H-NMR (400 MHz, *d*_6_-DMSO) spectrum of compound **7**; Figure S22: ^13^C-NMR (101 MHz, *d*_6_-DMSO) spectrum of compound **7**; Figure S23: Fullscan analysis of compound **7**; Figure S24: ^1^H-NMR (400 MHz, *d*_6_-DMSO) spectrum of compound **8a**; Figure S25: ^13^C-NMR (101 MHz, *d*_6_-DMSO) spectrum of compound **8a**; Figure S26: 2D NOESY (*d*_6_-DMSO) spectrum of compound **8a**; Figure S27: 2D HMBC (*d*_6_-DMSO) spectrum of compound **8a**; Figure S28: ^1^H-NMR (400 MHz, *d*_6_-DMSO) spectrum of compound **8b**; Figure S29: ^13^C-NMR (101 MHz, *d*_6_-DMSO) spectrum of compound **8b**; Figure S30: 2D NOESY (*d*_6_-DMSO) spectrum of compound **8b**; Figure S31: 2D HMBC (*d*_6_-DMSO) spectrum of compound **8b**; Figure S32: Fullscan analysis of isomers **8a** and **8b**; Figure S33: Fragmentation spectra of precursor ion *m*/*z* 261.1344 for isomers **8a** and **8b**; Figure S34: Fragmentation spectra of precursor ion m/z 215.0926 for isomers **8a** and **8b**.

## Sample Availability

Samples of the compounds **1**–**8** are available from the authors.
