# Supplementary material for: Regioselective Synthesis, Structural Characterization, and Antiproliferative Activity of Novel Tetra-Substituted Phenylaminopyrazole Derivatives
Source: Molecules. 2022 Sep 8;27(18):5814. doi: 10.3390/molecules27185814 (PMC9502416; doi:10.3390/molecules27185814)
Supplement: Supplementary file 1 [file molecules-27-05814-s001.zip › molecules-1887954-supplementary.pdf]

Supporting Information

Regioselective Synthesis, Structural Characterization, and Antiproliferative Activity of Novel Tetra-Substituted Phenylaminopyrazole Derivatives

Lusardi Matteo [1,†], Aldo Profumo [2,†], Chiara Rotolo [1], Erika Iervasi [2], Camillo Rosano [2], Andrea Spallarossa [1,*], Marco Ponassi[2]

[1]Department of Pharmacy, University of Genova, Viale Benedetto XV, 3, 16132 Genova, Italy;

[2]Proteomics and Mass Spectrometry Unit, IRCCS Ospedale Policlinico San Martino, L.go R. Benzi 10, 16132 Genova, Italy;

[†] Equal contribution

* Corresponding author (andrea.spallarossa@unige.it)

Table of contents

Figure S1. ^{1}H-NMR (400 MHz, d_6-DMSO) spectrum of compound **1**

Figure S2. ^{13}C-NMR (101 MHz, d_6-DMSO) spectrum of compound **1**

Figure S3. 2D NOESY (d_6-DMSO) spectrum of compound **1**

Figure S4. 2D HMBC (d_6-DMSO) spectrum of compound **1**

Figure S5. Fullscan analysis of compound **1**

Figure S6. ^{1}H-NMR (400 MHz, d_6-DMSO) spectrum of compound **2**

Figure S7. ^{13}C-NMR (101 MHz, d_6-DMSO) spectrum of compound **2**

Figure S8. Fullscan analysis of compound **2**

Figure S9. ^{1}H-NMR (400 MHz, d_6-DMSO) spectrum of compound **3**

Figure S10. ^{13}C-NMR (101 MHz, d_6-DMSO) spectrum of compound **3**

Figure S11. Fullscan analysis of compound **3**

Figure S12. ^{1}H-NMR (400 MHz, d_6-DMSO) spectrum of compound **4**

Figure S13. ^{13}C-NMR (101 MHz, d_6-DMSO) spectrum of compound **4**

Figure S14. Fullscan analysis of compound **4**

Figure S15. ^{1}H-NMR (400 MHz, d_6-DMSO) spectrum of compound **5**

Figure S16. ^{13}C-NMR (101 MHz, d_6-DMSO) spectrum of compound **5**

Figure S17. Fullscan analysis of compound **5**

Figure S18. ^{1}H-NMR (400 MHz, d_6-DMSO) spectrum of compound **6**

Figure S19. ^{13}C-NMR (101 MHz, d_6-DMSO) spectrum of compound **6**

Figure S20. Fullscan analysis of compound **6**

Figure S21. ^{1}H-NMR (400 MHz, d_6-DMSO) spectrum of compound **7**

Figure S22. ^{13}C-NMR (101 MHz, d_6-DMSO) spectrum of compound **7**

Figure S23. Fullscan analysis of compound **7**

Figure S24. ^{1}H-NMR (400 MHz, d_6-DMSO) spectrum of compound **8a**

Figure S25. ^{13}C-NMR (101 MHz, d_6-DMSO) spectrum of compound **8a**

Figure S26. 2D NOESY (d_6-DMSO) spectrum of compound **8a**

Figure S27. 2D HMBC (d_6-DMSO) spectrum of compound **8a**

Figure S28. ^{1}H-NMR (400 MHz, d_6-DMSO) spectrum of compound **8b**

Figure S29. ^{13}C-NMR (101 MHz, d_6-DMSO) spectrum of compound **8b**

Figure S30. 2D NOESY (d_6-DMSO) spectrum of compound **8b**

Figure S31. 2D HMBC (d_6-DMSO) spectrum of compound **8b**

Figure S32. Fullscan analysis of isomers **8a** and **8b**

Figure S33. Fragmentation spectra of precursor ion m/z 261.1344 for isomers **8a** and **8b**.

Figure S34. Fragmentation spectra of precursor ion m/z 215.0926 for isomers **8a** and **8b**

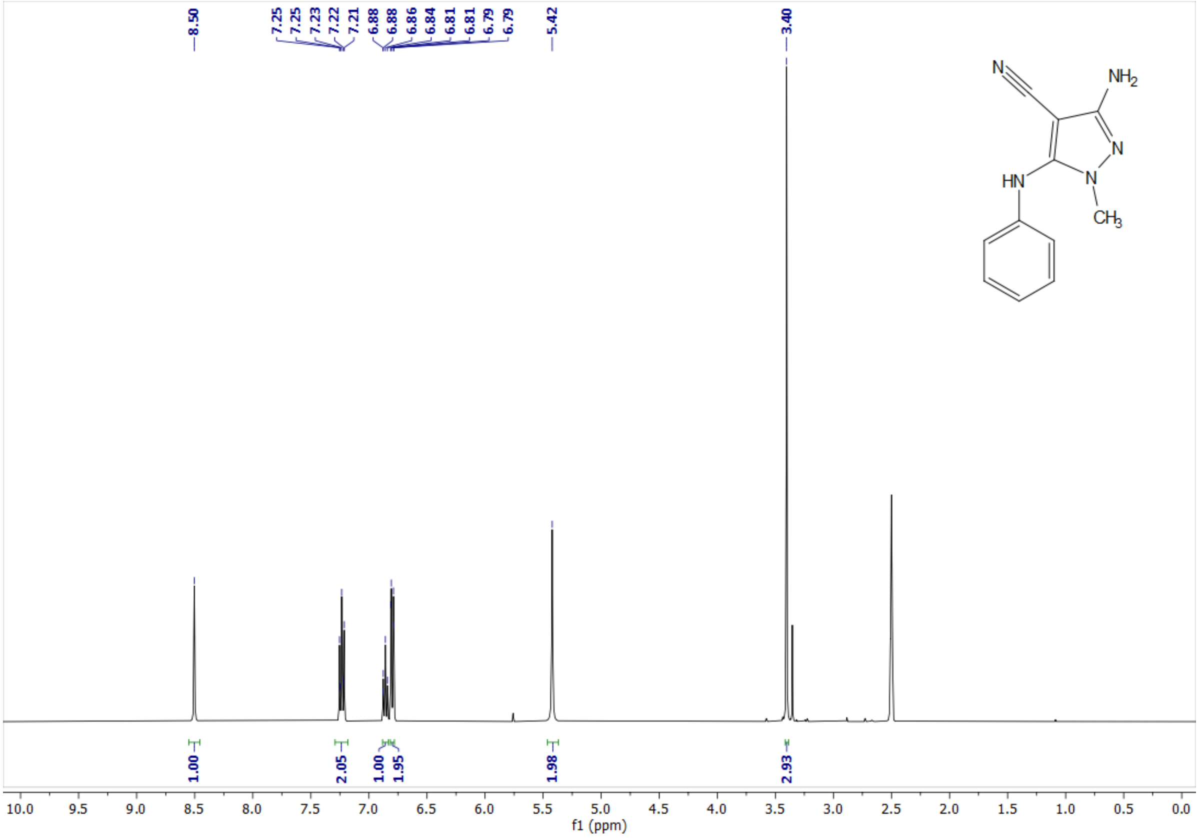

Figure S1. ^{1}H-NMR (400 MHz, d_6-DMSO) spectrum of compound **1**

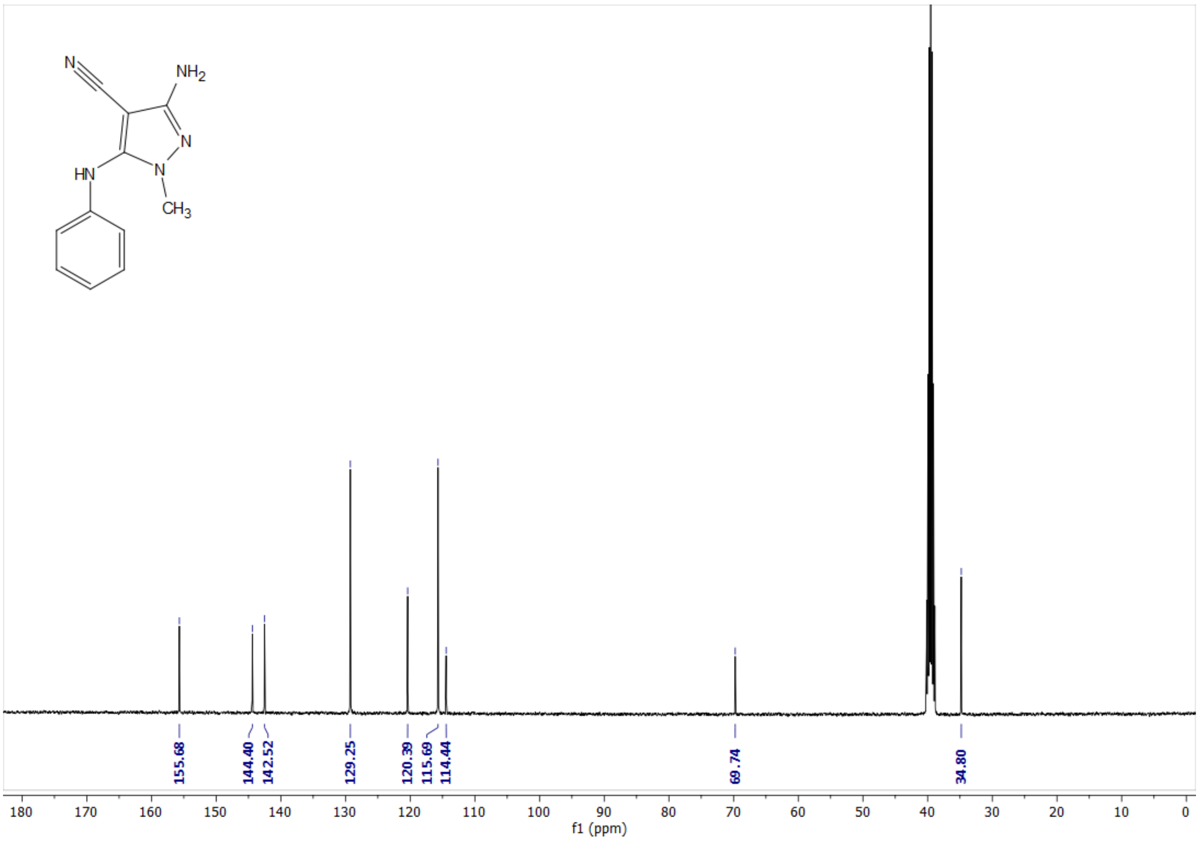

Figure S2. ^{13}C-NMR (101 MHz, d_6-DMSO) spectrum of compound **1**

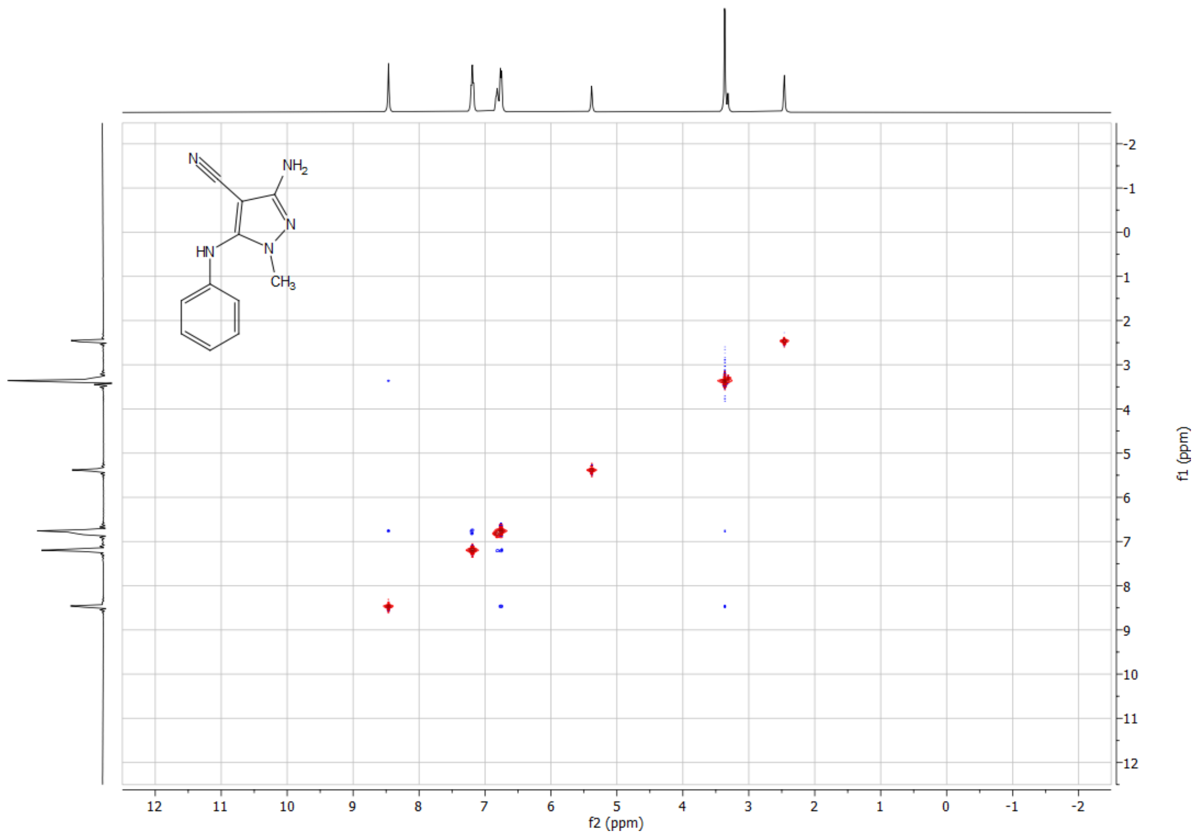

Figure S3. 2D NOESY (d_6-DMSO) spectrum of compound **1**

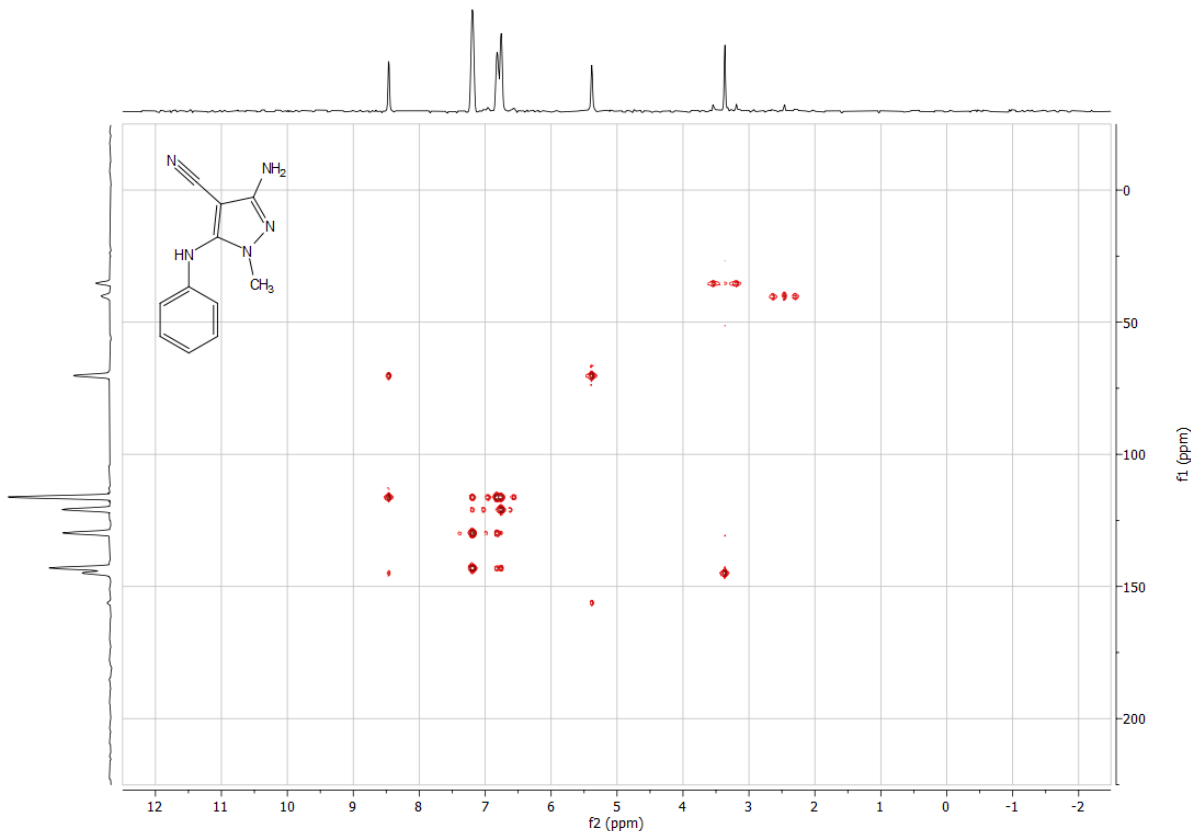

Figure S4. 2D HMBC (d_6-DMSO) spectrum of compound **1**

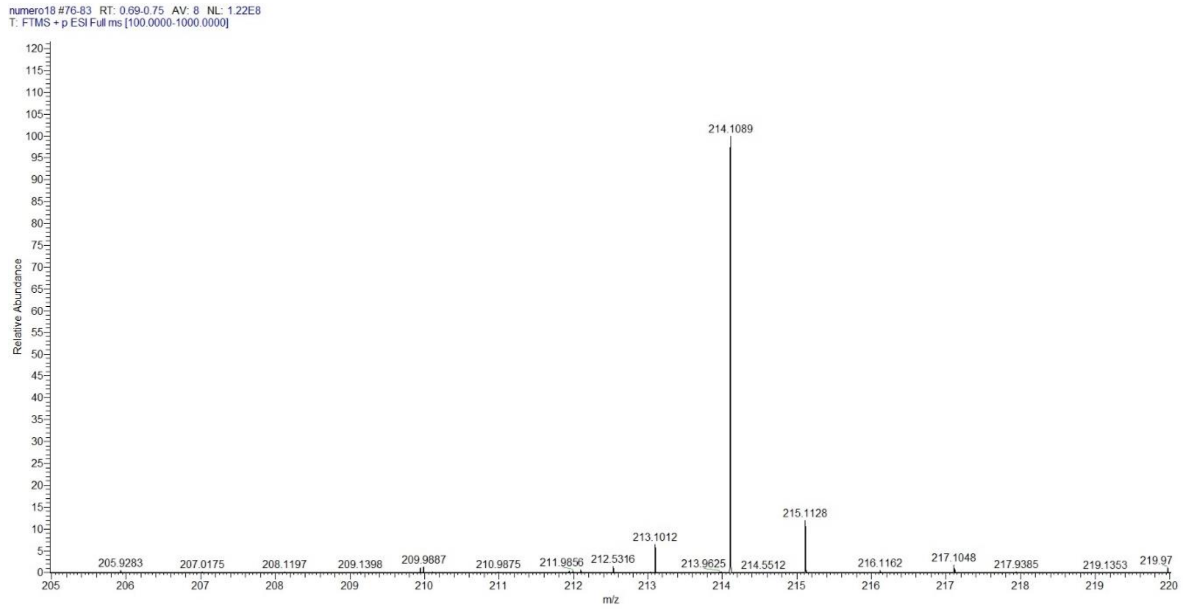

Figure S5. Fullscan analysis of compound **1**

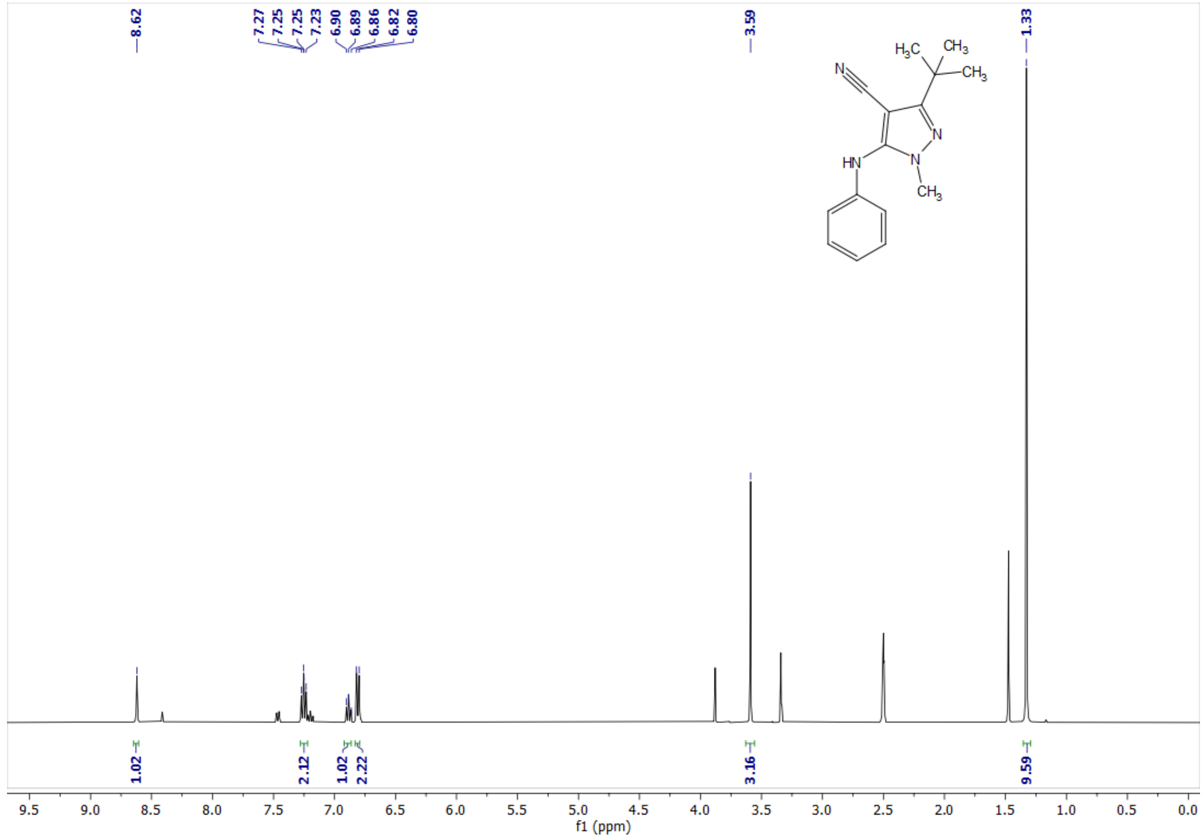

Figure S6. ^{1}H-NMR (400 MHz, d_6-DMSO) spectrum of compound **2**

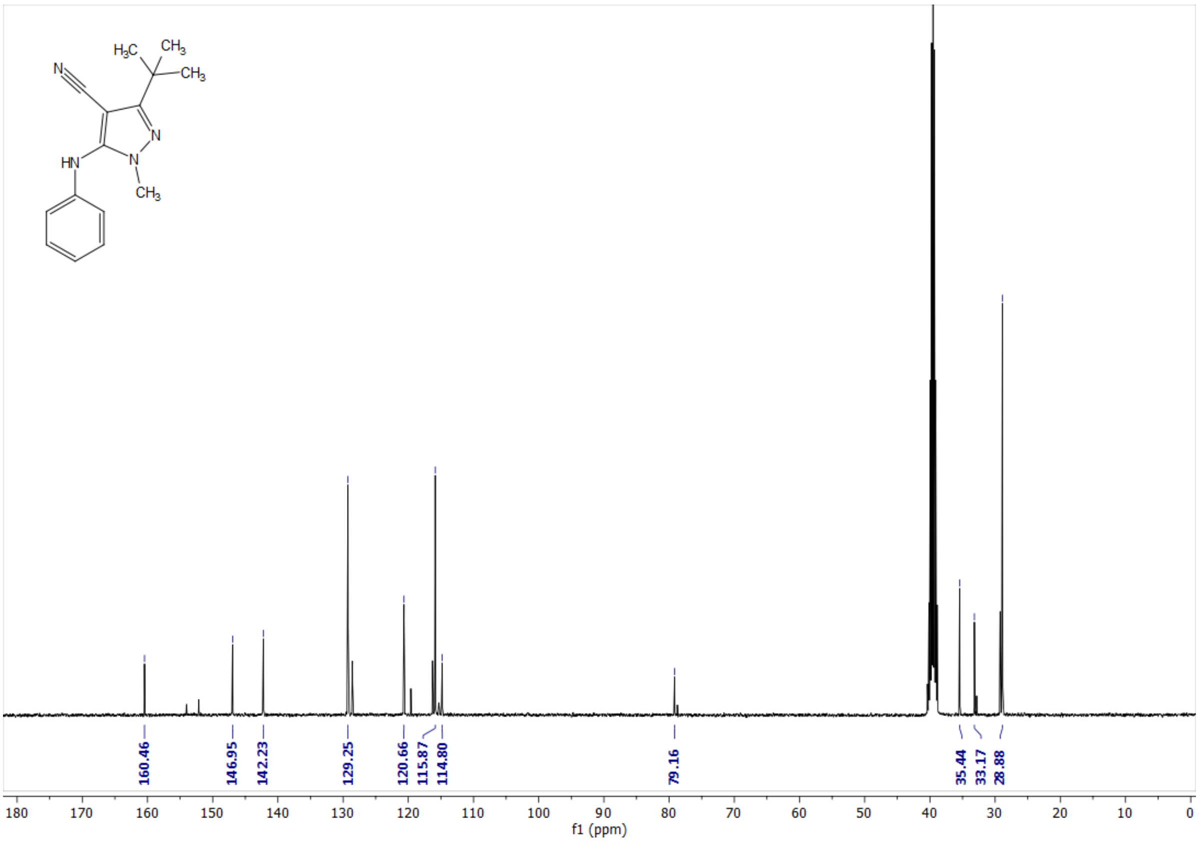

Figure S7. ^{13}C-NMR (101 MHz, d_6-DMSO) spectrum of compound **2**

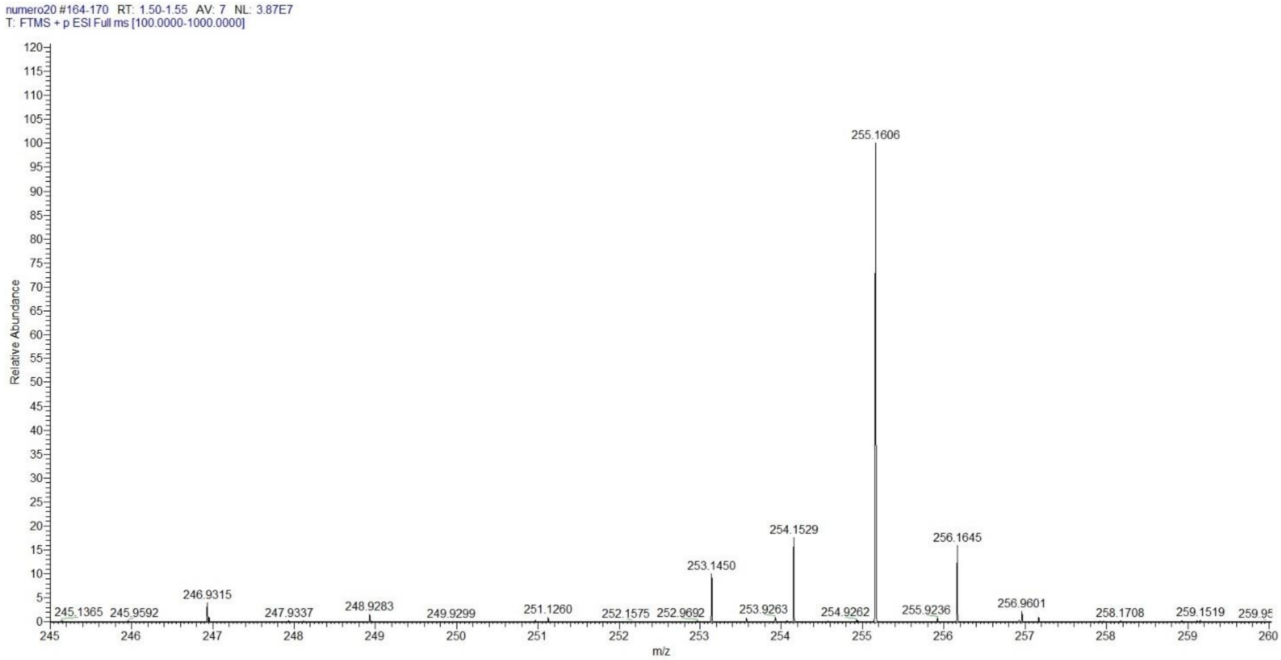

Figure S8. Fullscan analysis of compound **2**

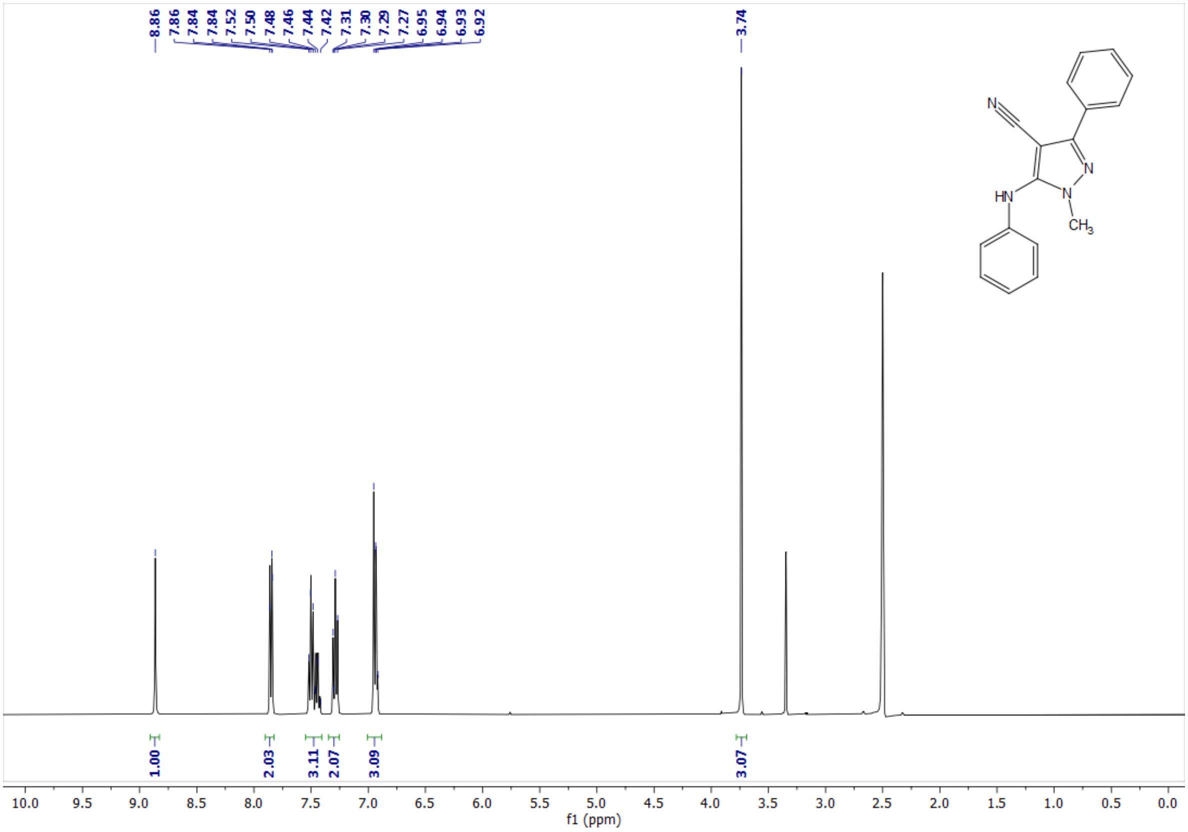

Figure S9. ^{1}H-NMR (400 MHz, d_6-DMSO) spectrum of compound **3**

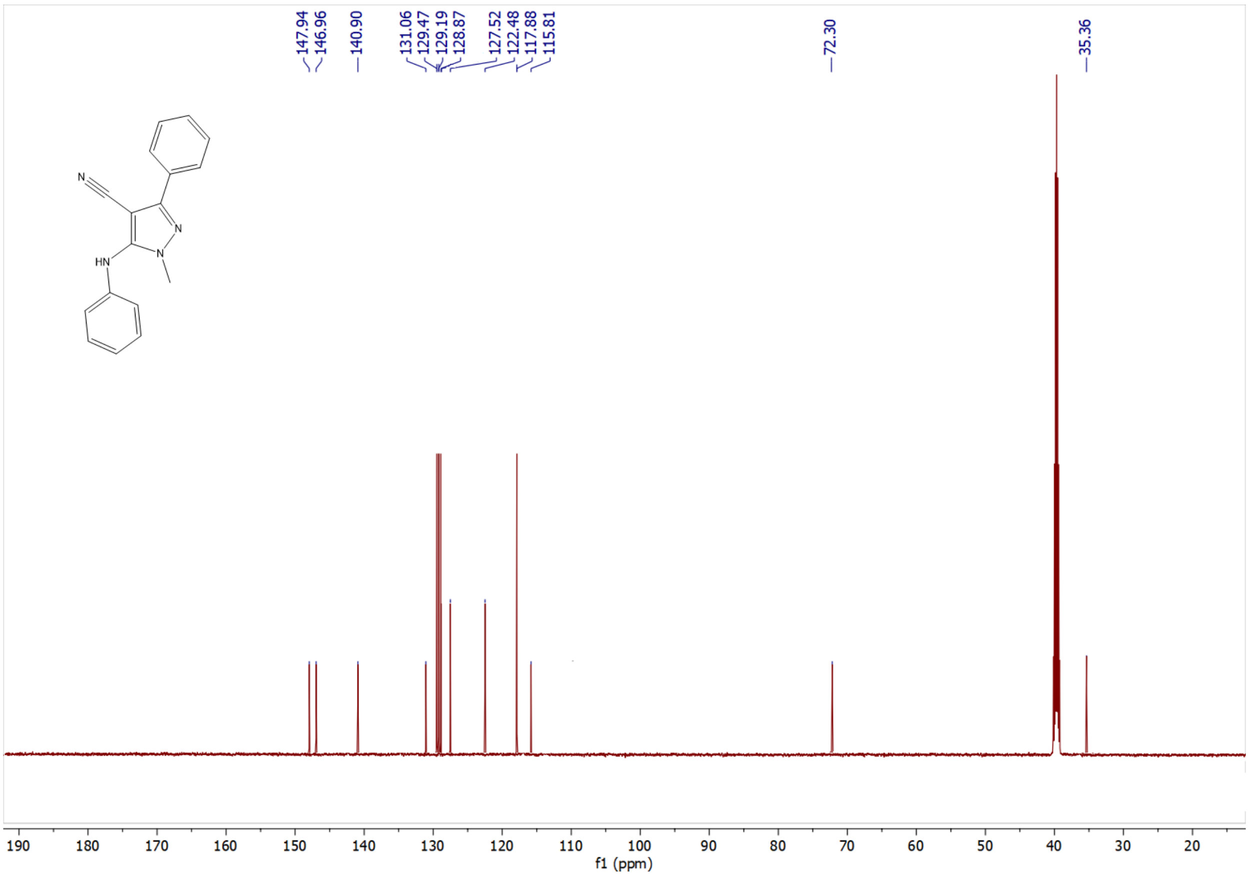

Figure S10. ^{13}C-NMR (101 MHz, d_6-DMSO) spectrum of compound **3**

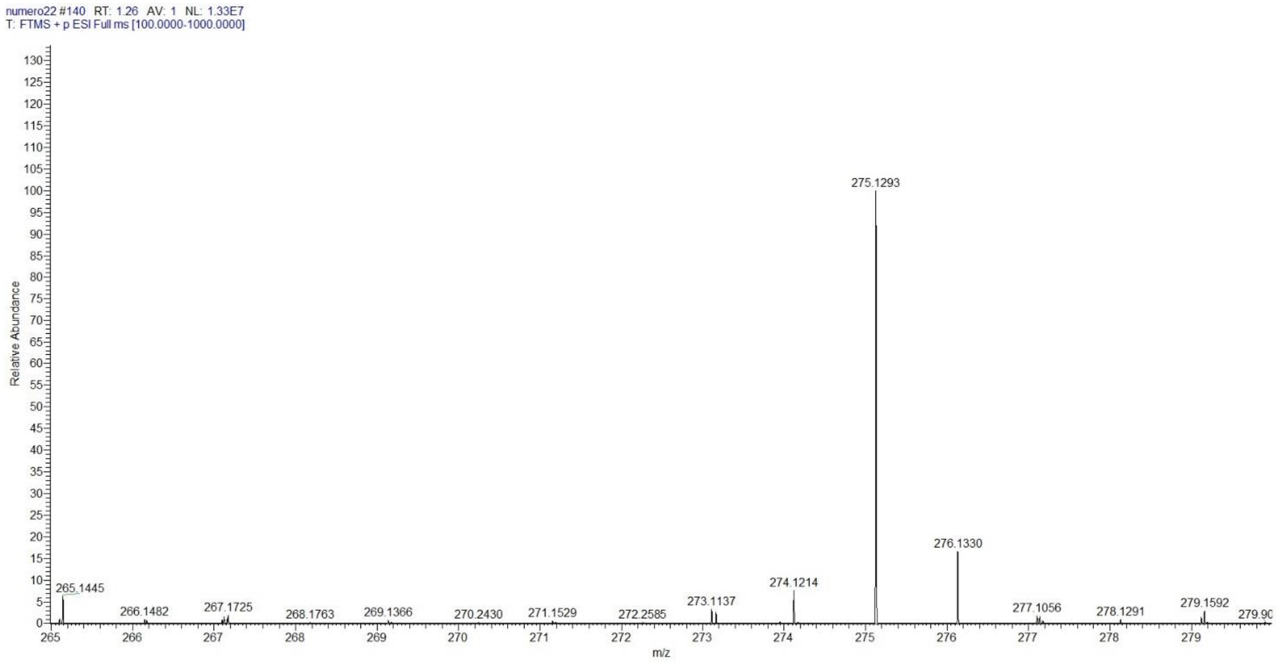

Figure S11. Fullscan analysis of compound **3**

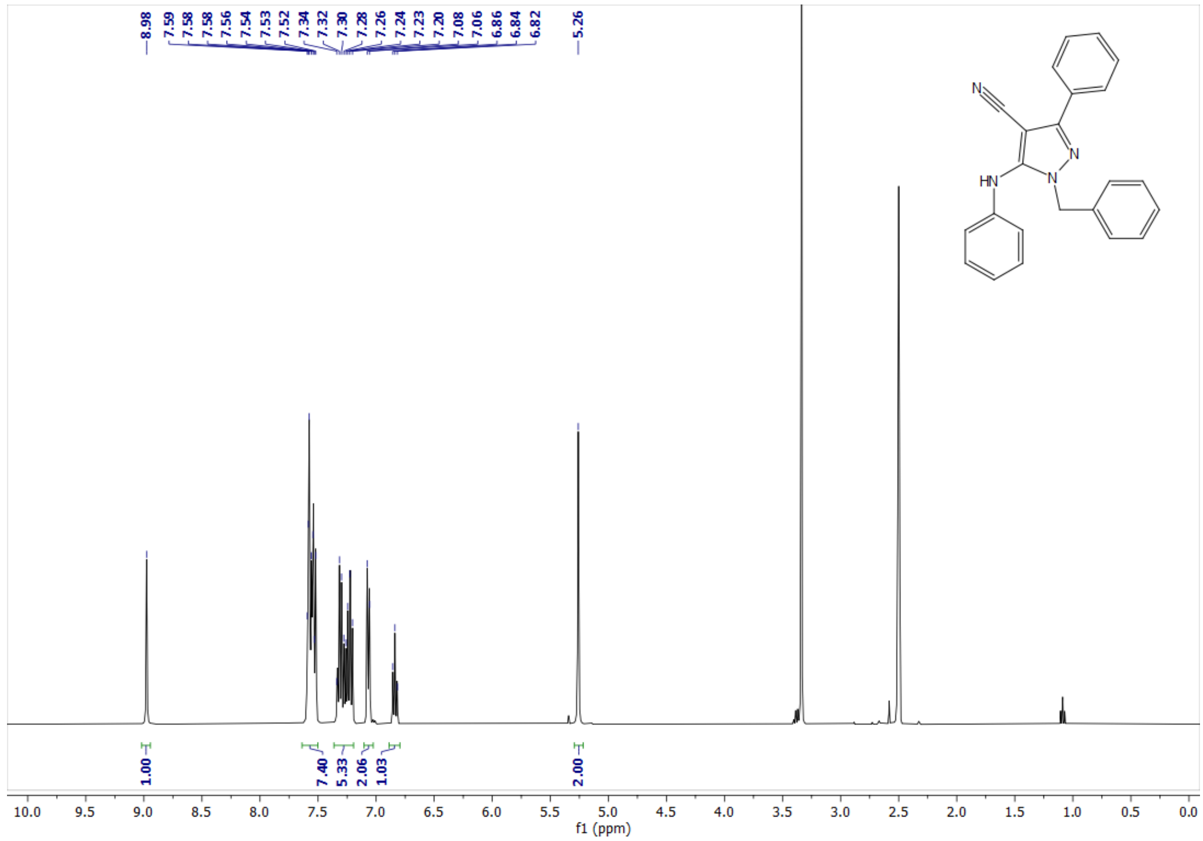

Figure S12. ^{1}H-NMR (400 MHz, d_6-DMSO) spectrum of compound **4**

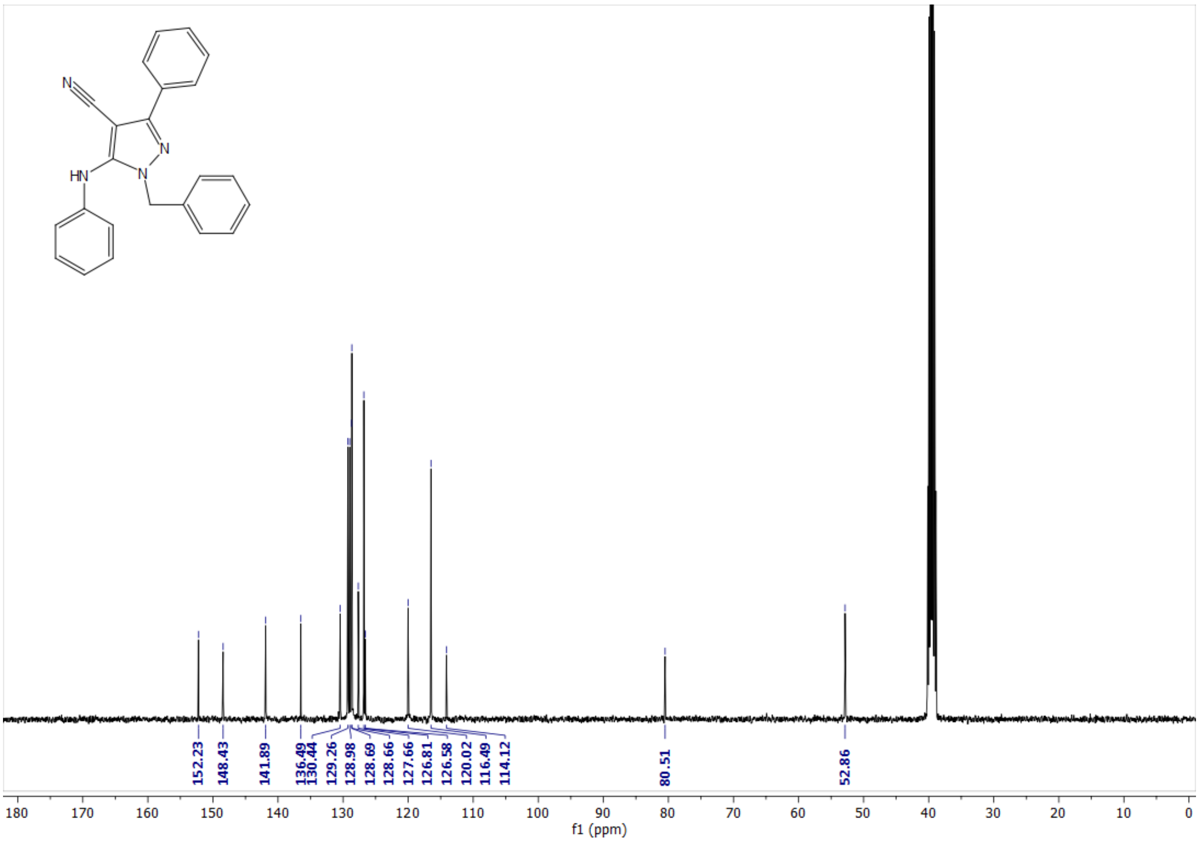

Figure S13. ^{13}C-NMR (101 MHz, d_6-DMSO) spectrum of compound **4**

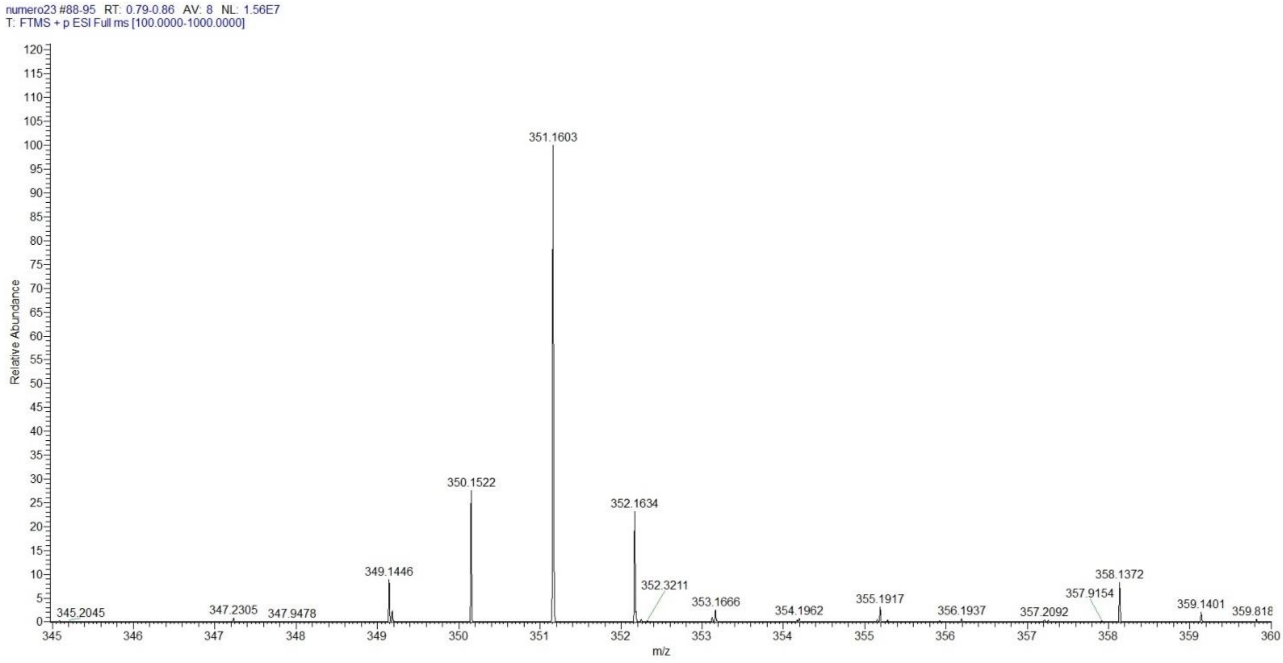

Figure S14. Fullscan analysis of compound **4**

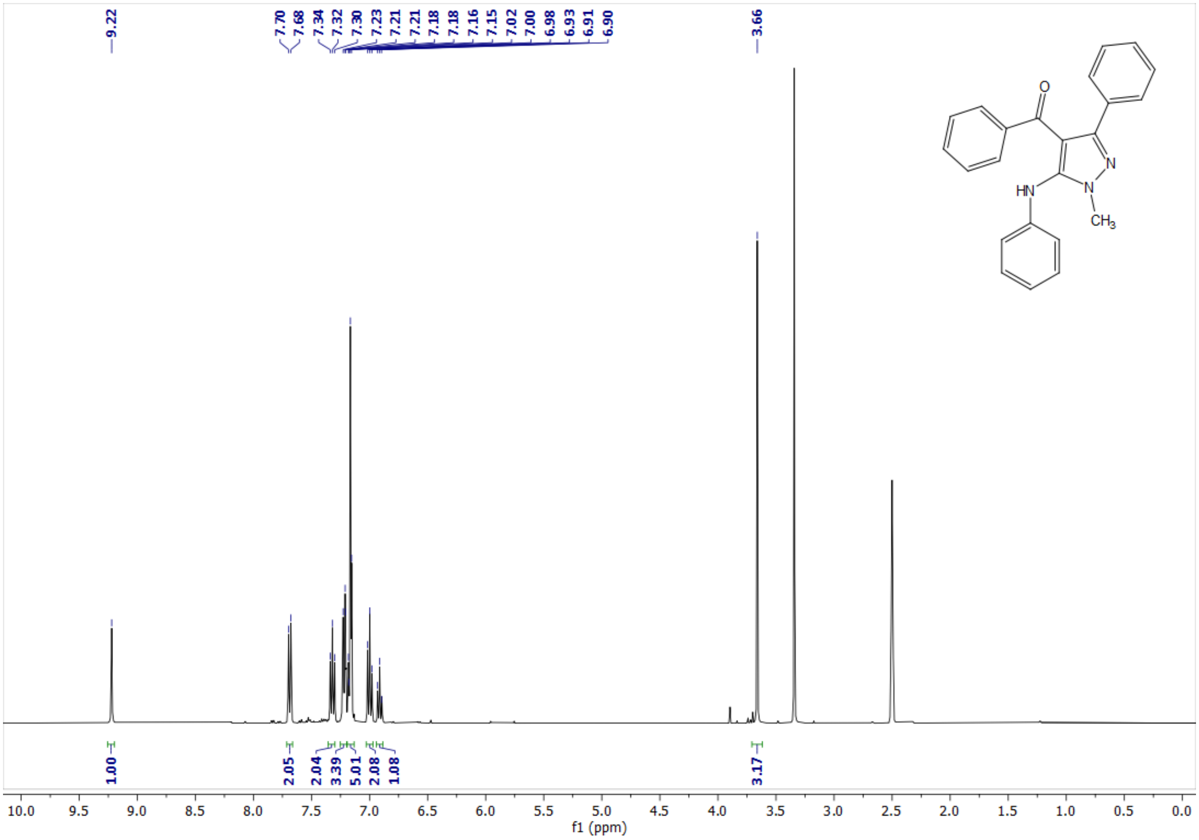

Figure S15. ^{1}H-NMR (400 MHz, d_6-DMSO) spectrum of compound **5**

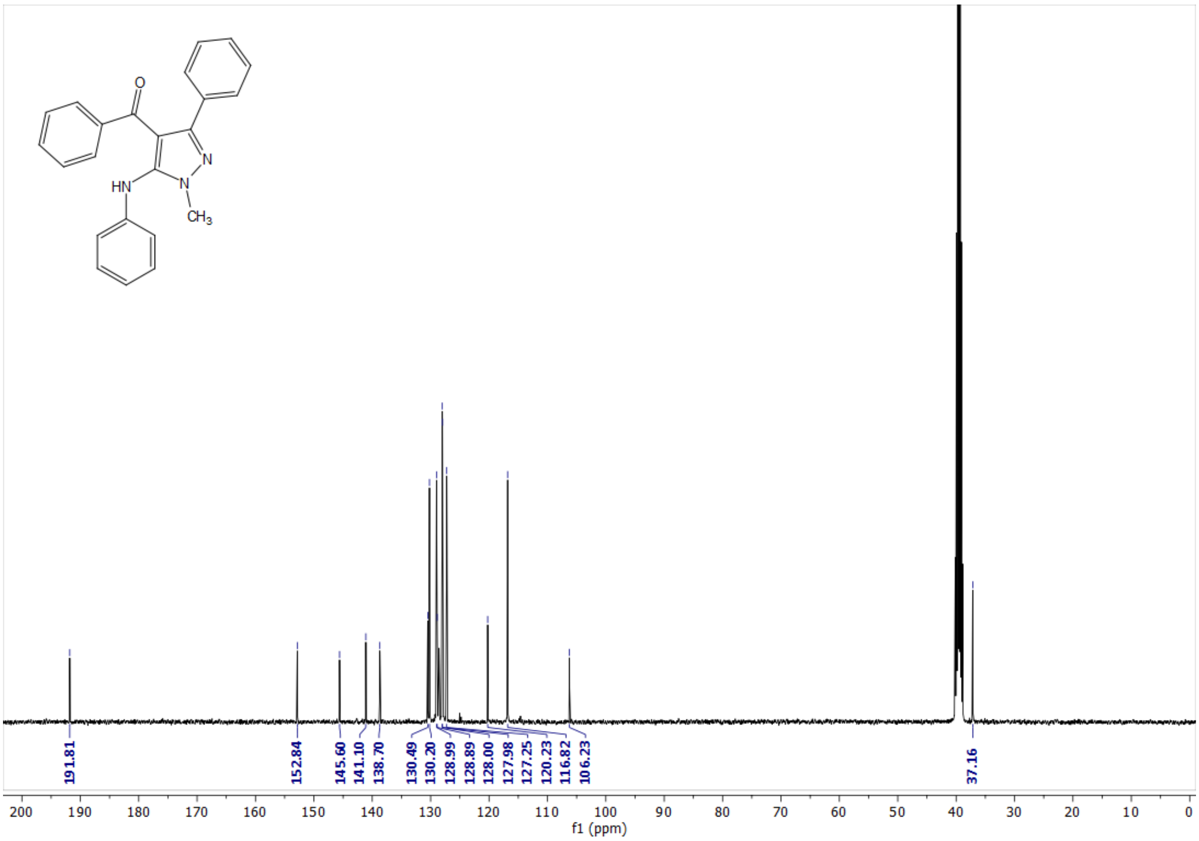

Figure S16. ^{13}C-NMR (101 MHz, d_6-DMSO) spectrum of compound **5**

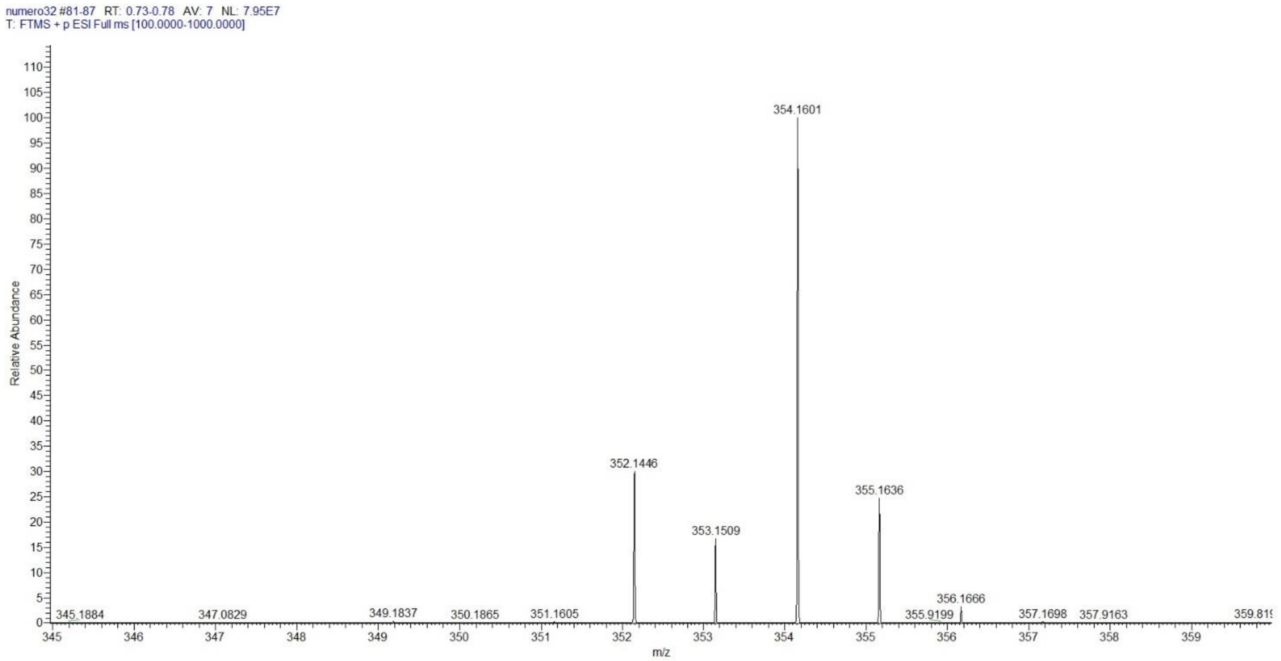

Figure S17. Fullscan analysis of compound **5**

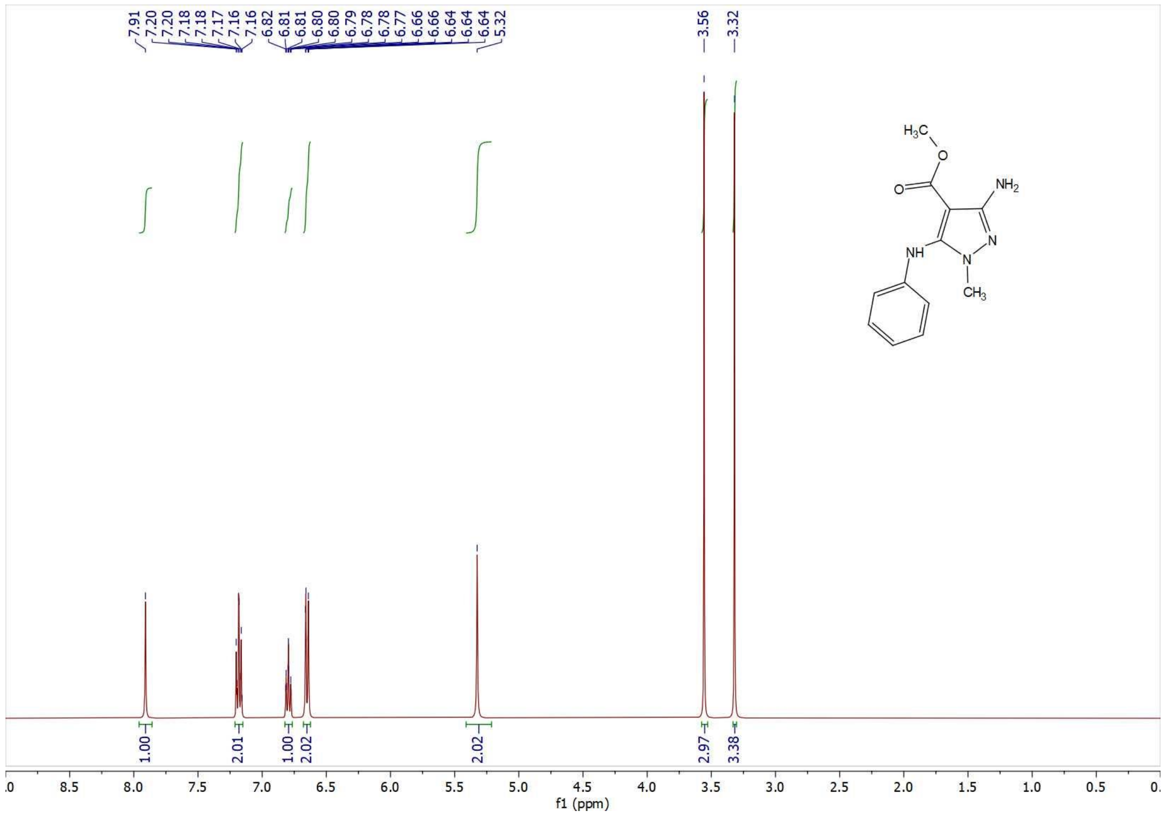

Figure S18. ^{1}H-NMR (400 MHz, d_6-DMSO) spectrum of compound **6**

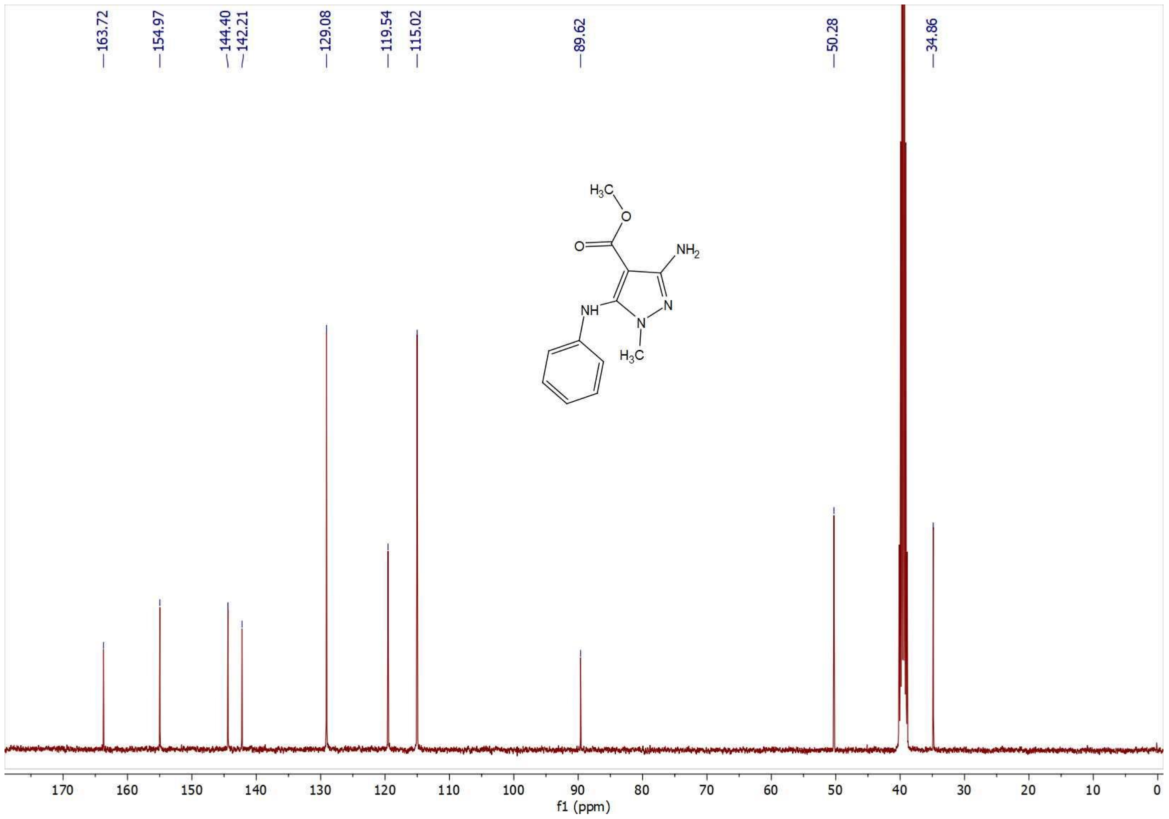

Figure S19. ^{13}C-NMR (101 MHz, d_6-DMSO) spectrum of compound **6**

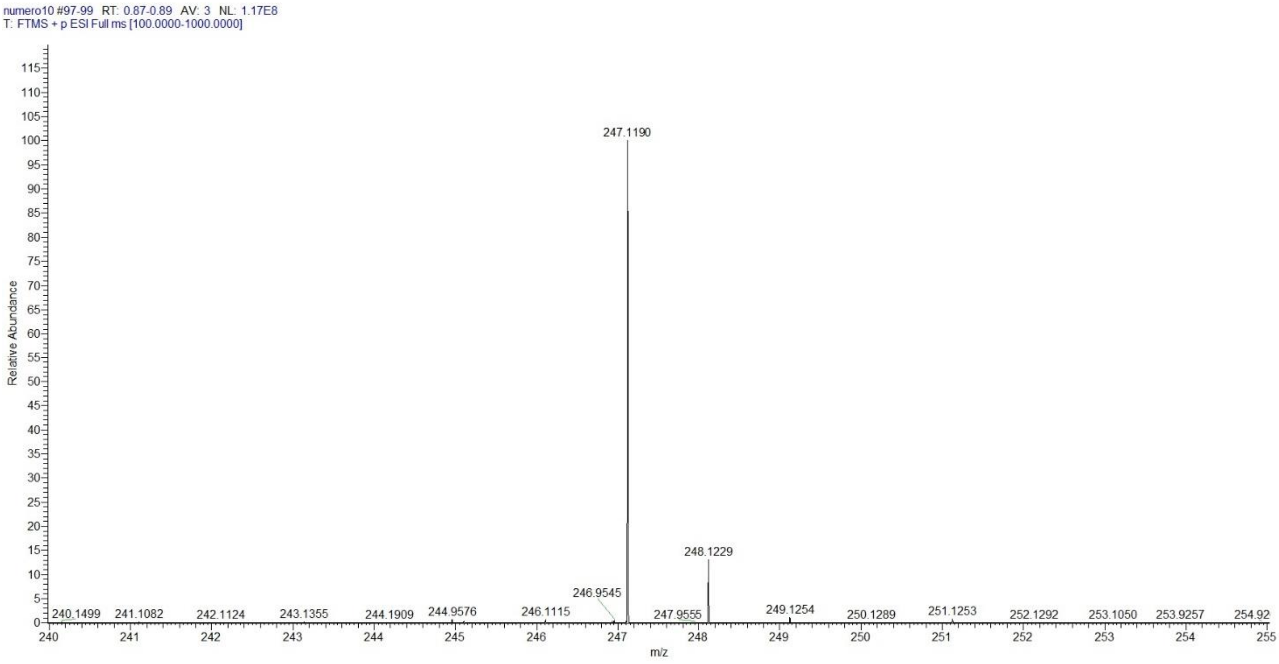

Figure S20. Fullscan analysis of compound **6**

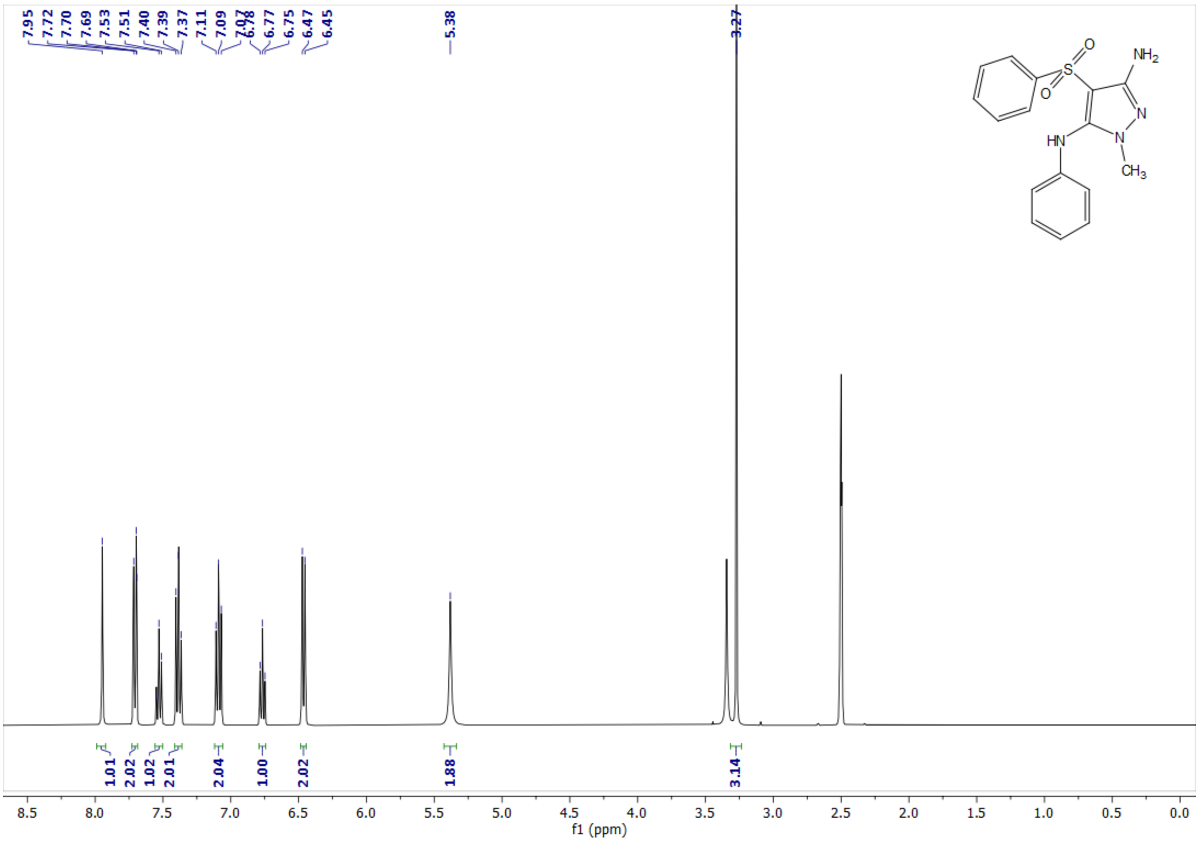

Figure S21. ^{1}H-NMR (400 MHz, d_6-DMSO) spectrum of compound **7**

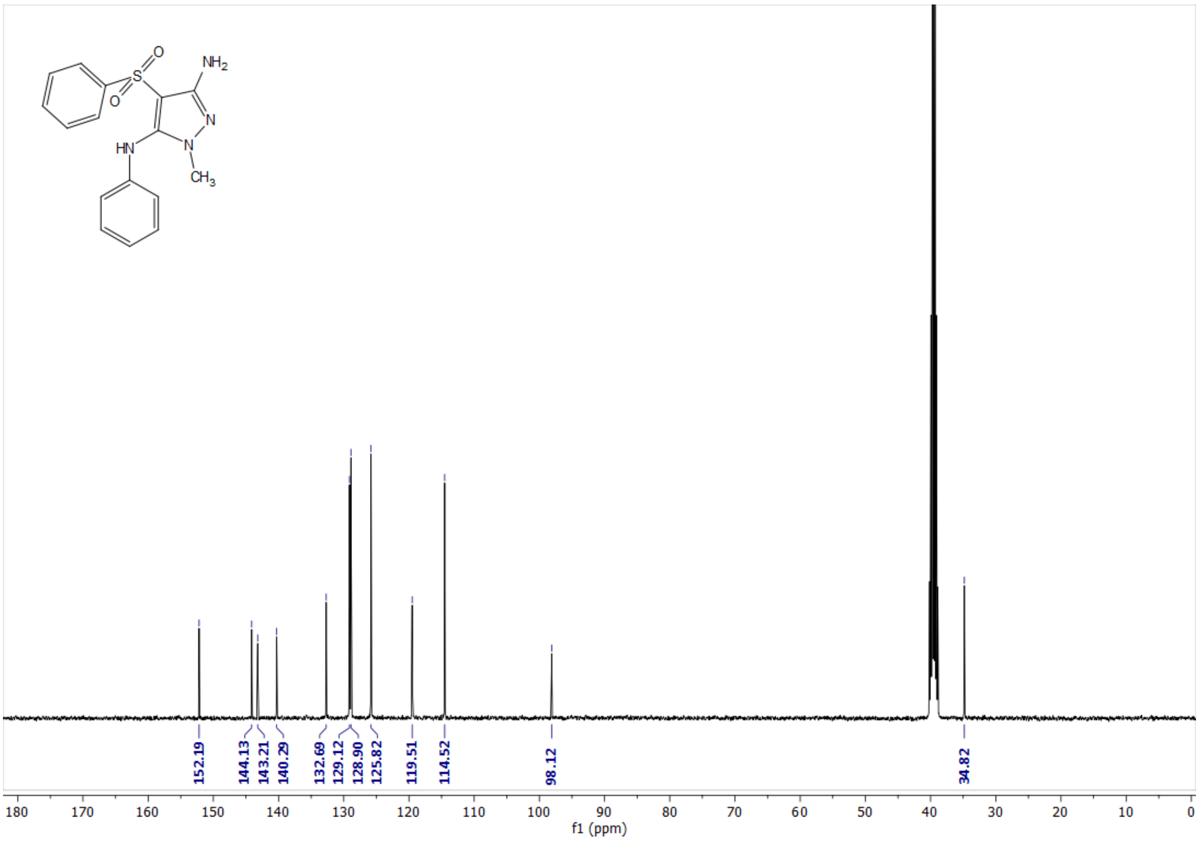

Figure S22. ^{13}C-NMR (101 MHz, d_6-DMSO) spectrum of compound **7**

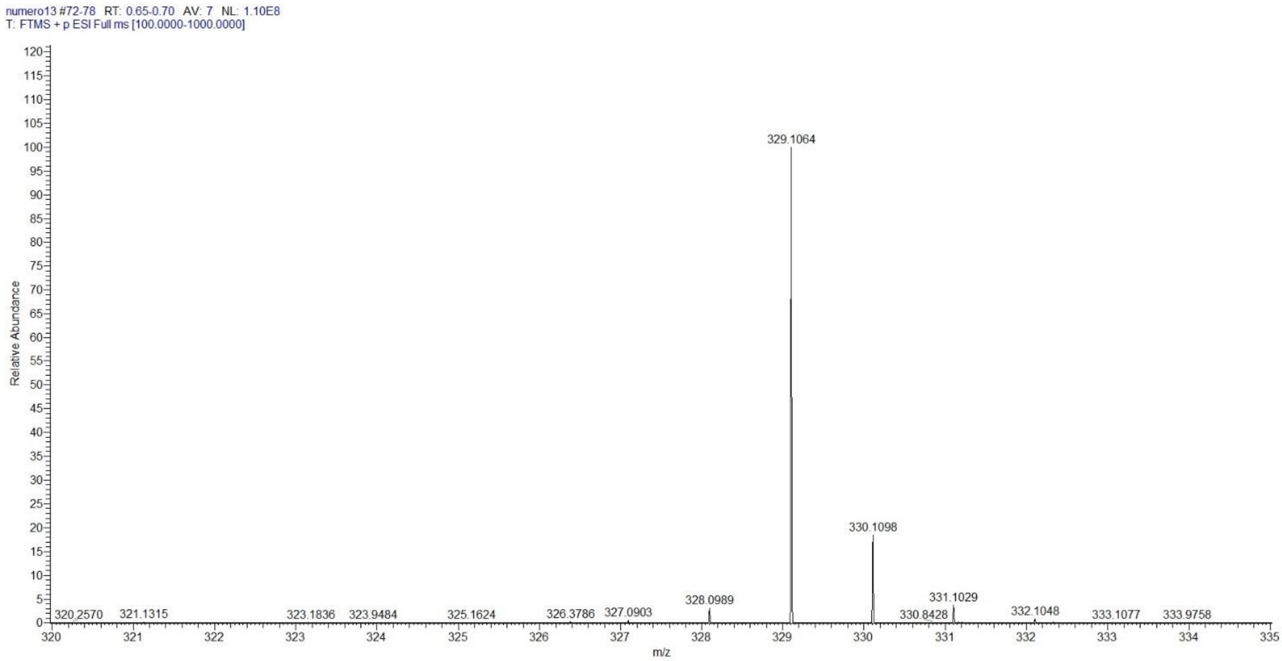

Figure S23. Fullscan analysis of compound **7**

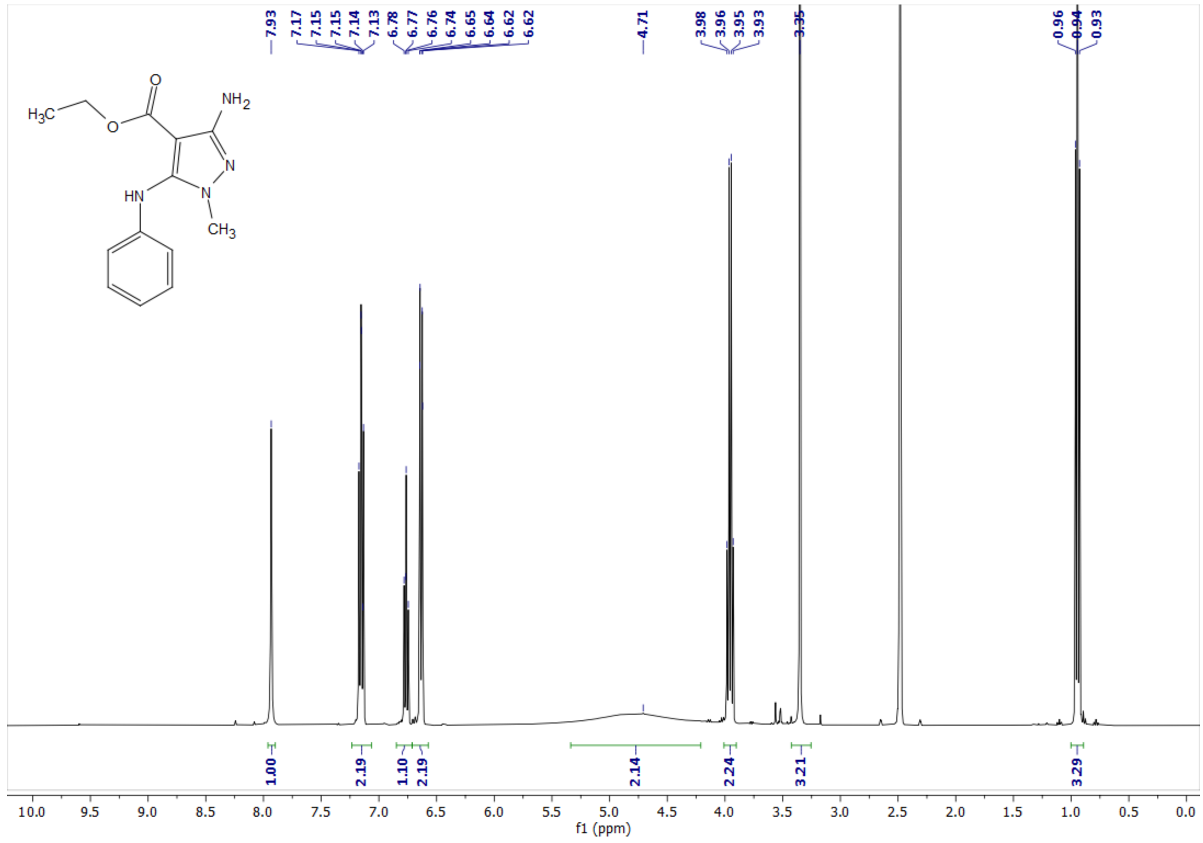

Figure S24. ^{1}H-NMR (400 MHz, d_6-DMSO) spectrum of compound **8a**

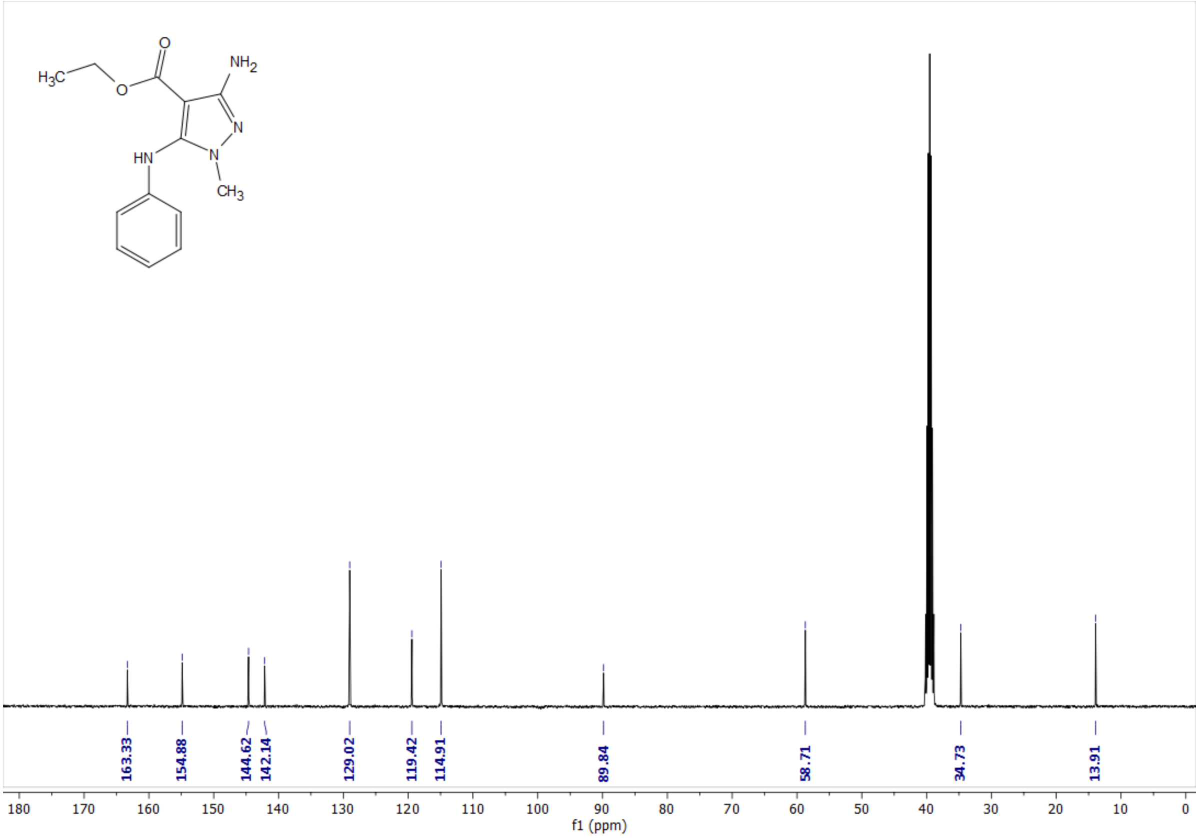

Figure S25. ^{13}C-NMR (101 MHz, d_6-DMSO) spectrum of compound **8a**

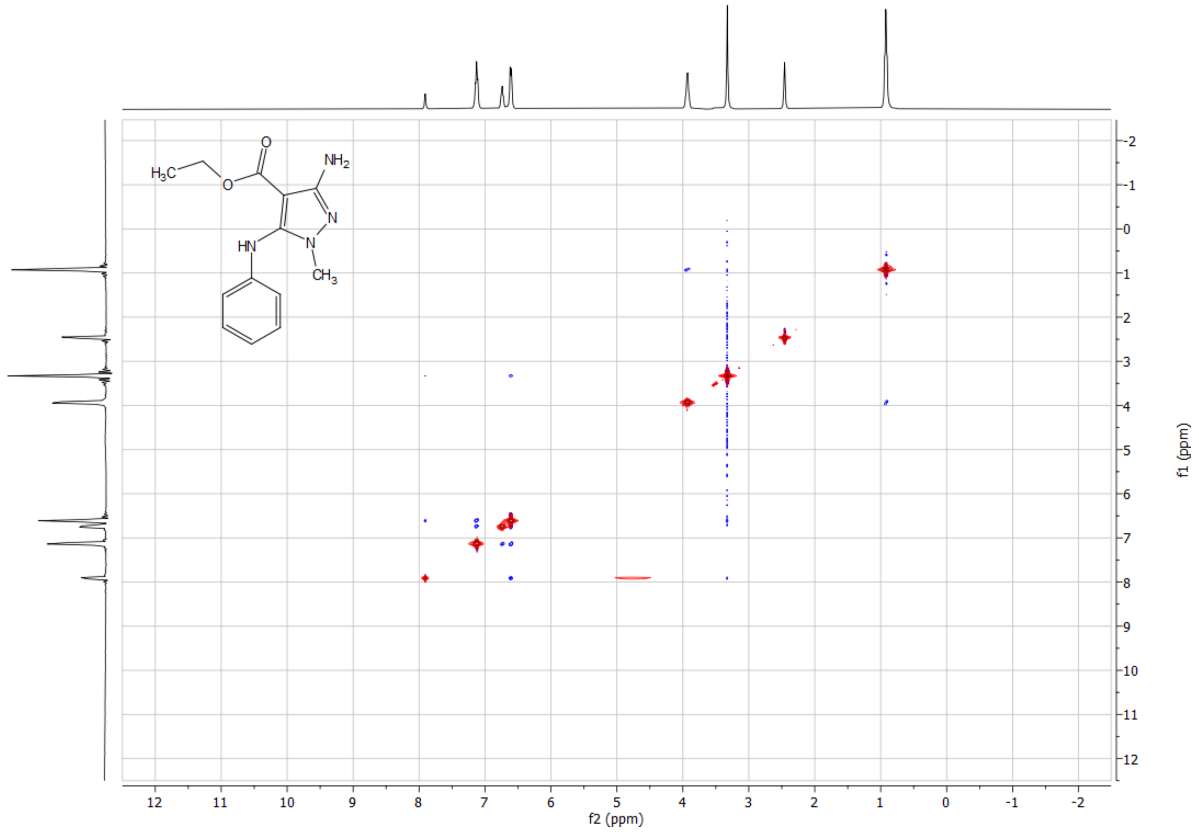

Figure S26. 2D NOESY (d_6-DMSO) spectrum of compound **8a**

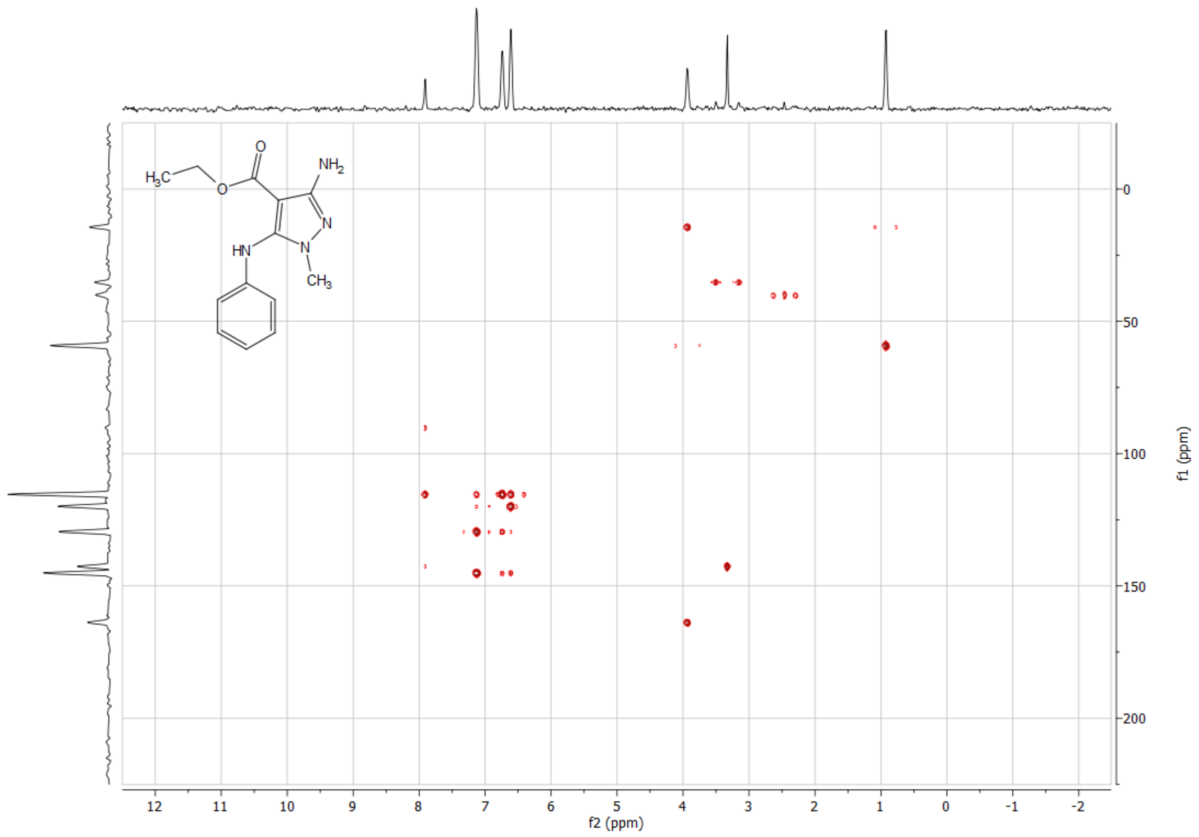

Figure S27. 2D HMBC (d_6-DMSO) spectrum of compound **8a**

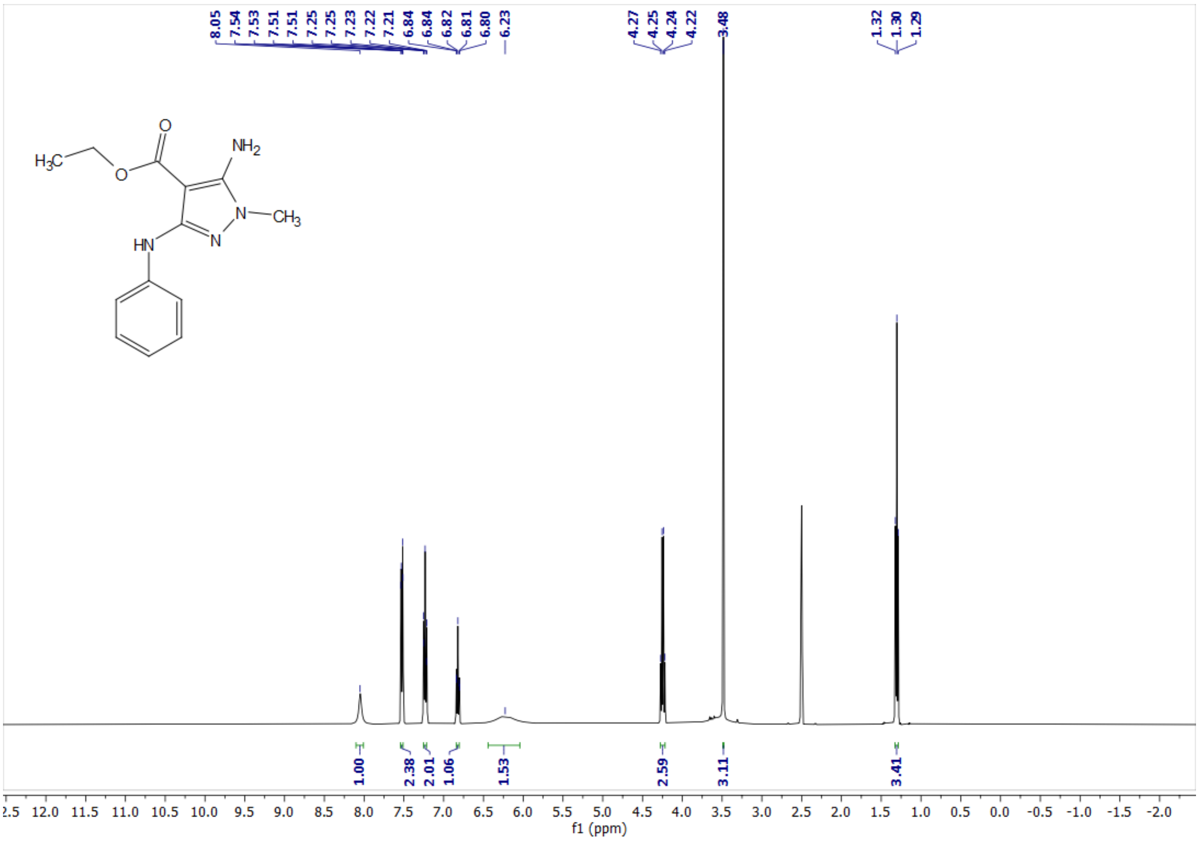

Figure S28. ^{1}H-NMR (400 MHz, d_6-DMSO) spectrum of compound **8b**

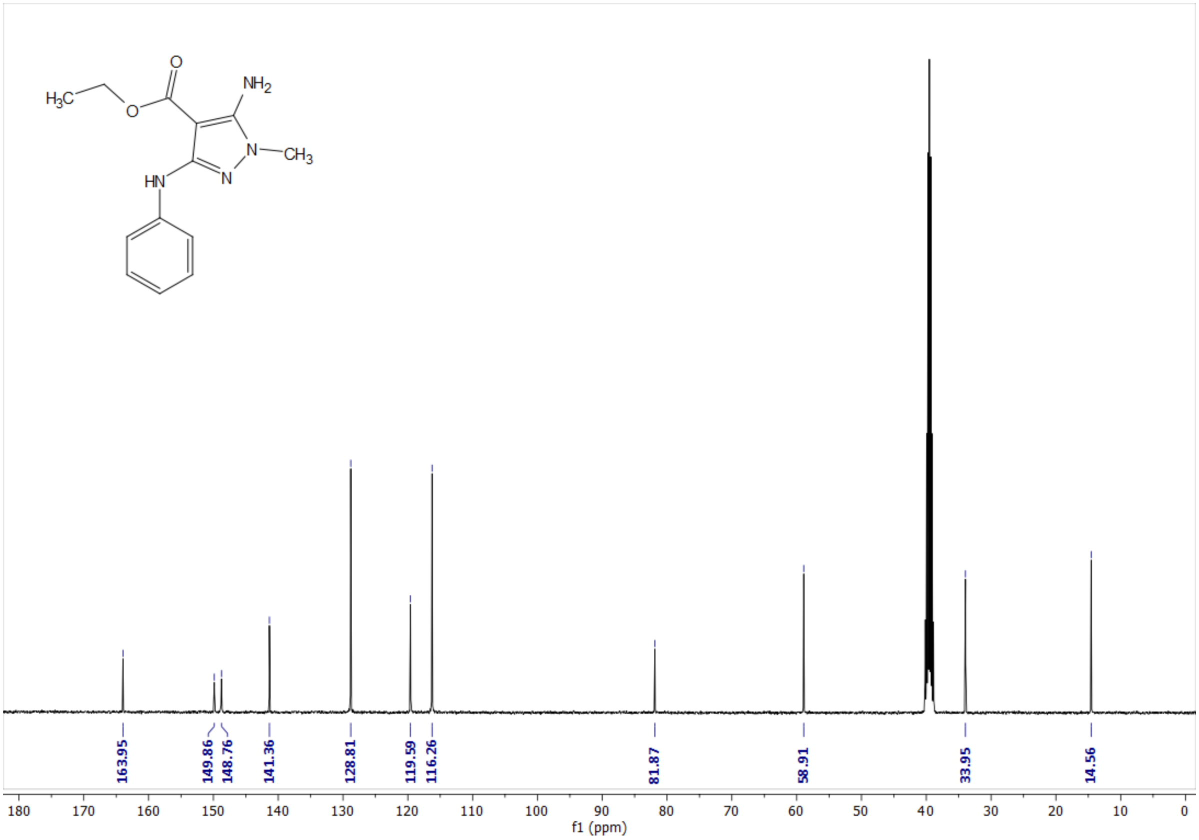

Figure S29. ^{13}C-NMR (101 MHz, d_6-DMSO) spectrum of compound **8b**

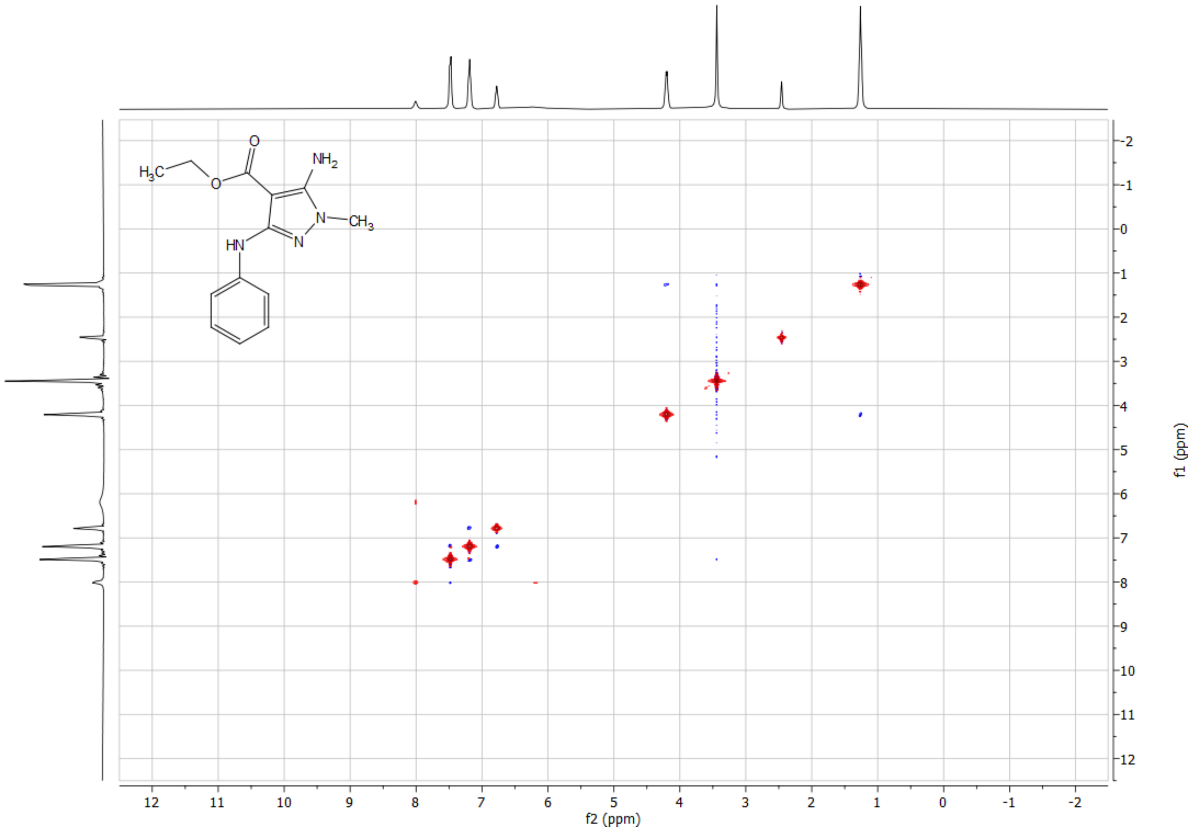

Figure S30. 2D NOESY (d_6-DMSO) spectrum of compound **8b**

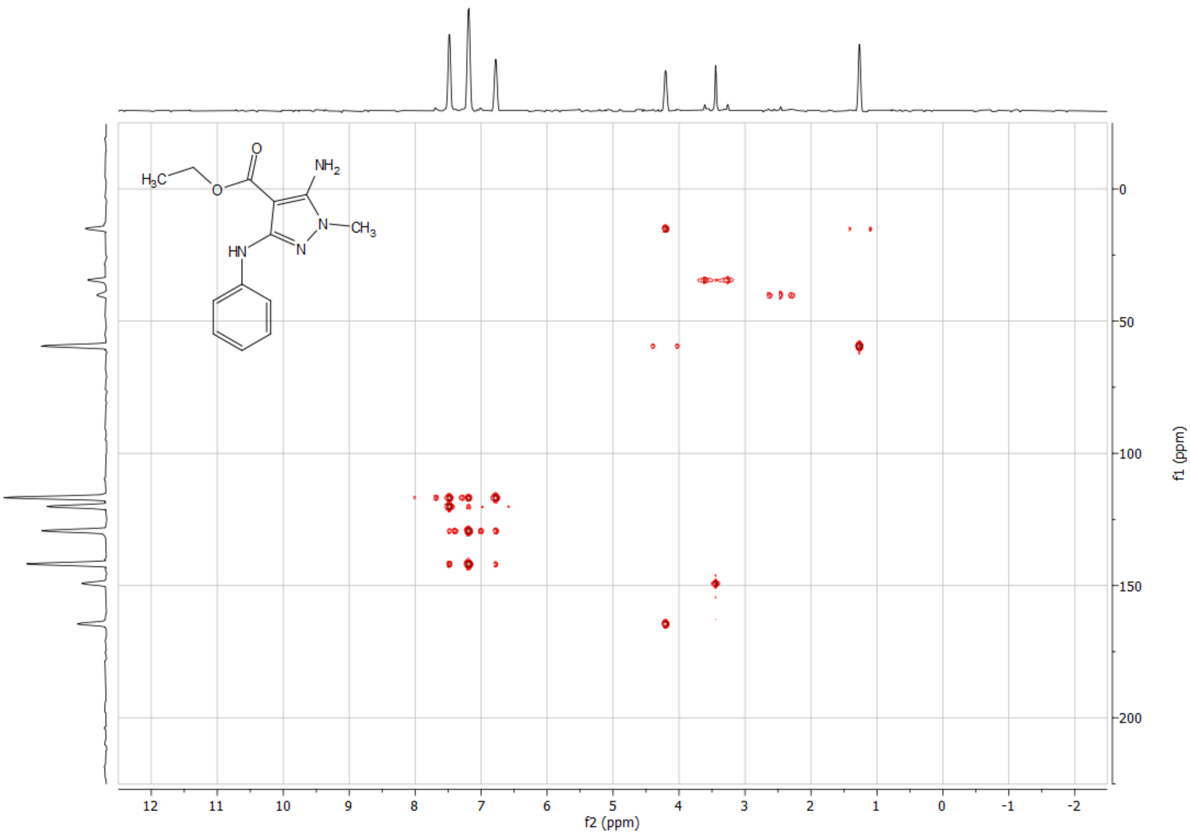

Figure S31. 2D HMBC (d_6-DMSO) spectrum of compound **8b**

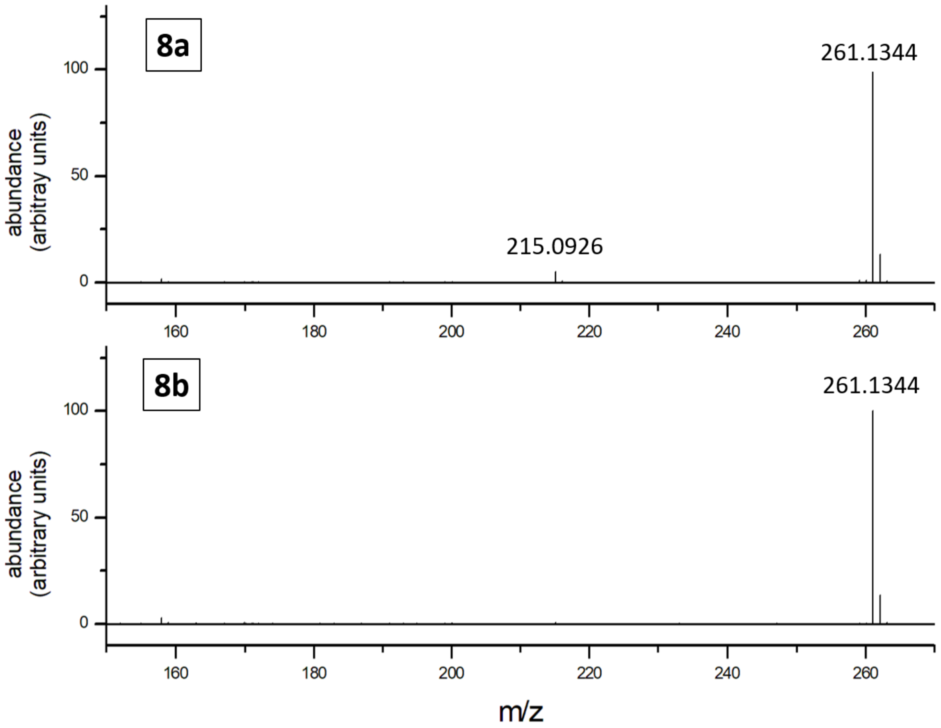

Figure S32. Fullscan analysis of isomers **8a** and **8b**

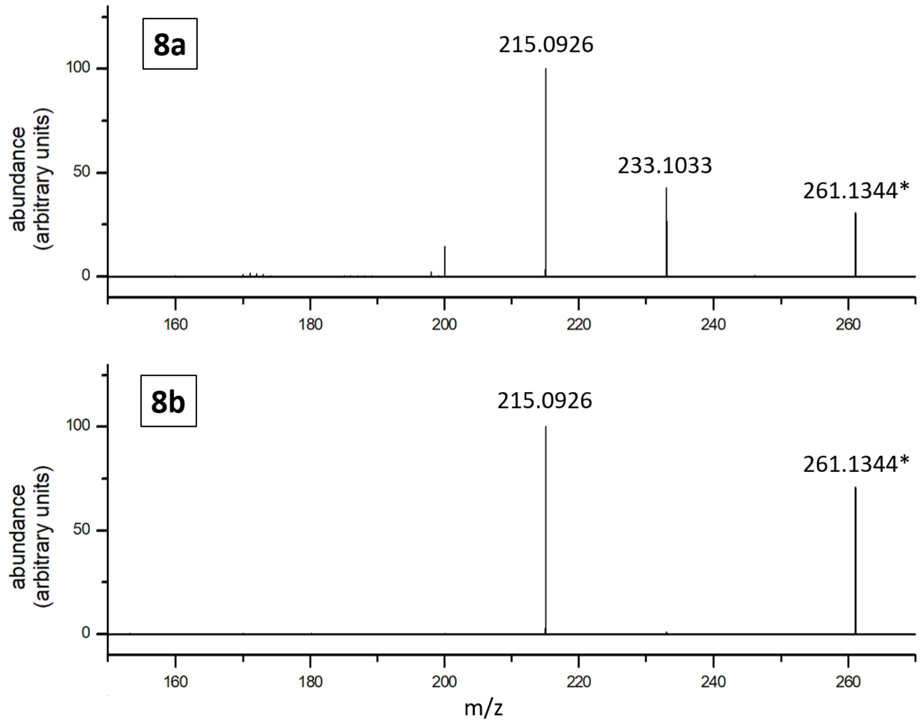

Figure S33. Fragmentation spectra of precursor ion m/z 261.1344 for isomers **8a** and **8b**. The precursor ion is marked by an asterisk. In sample **8a** a transient intermediate fragment (m/z 233.1033) is evidenced.

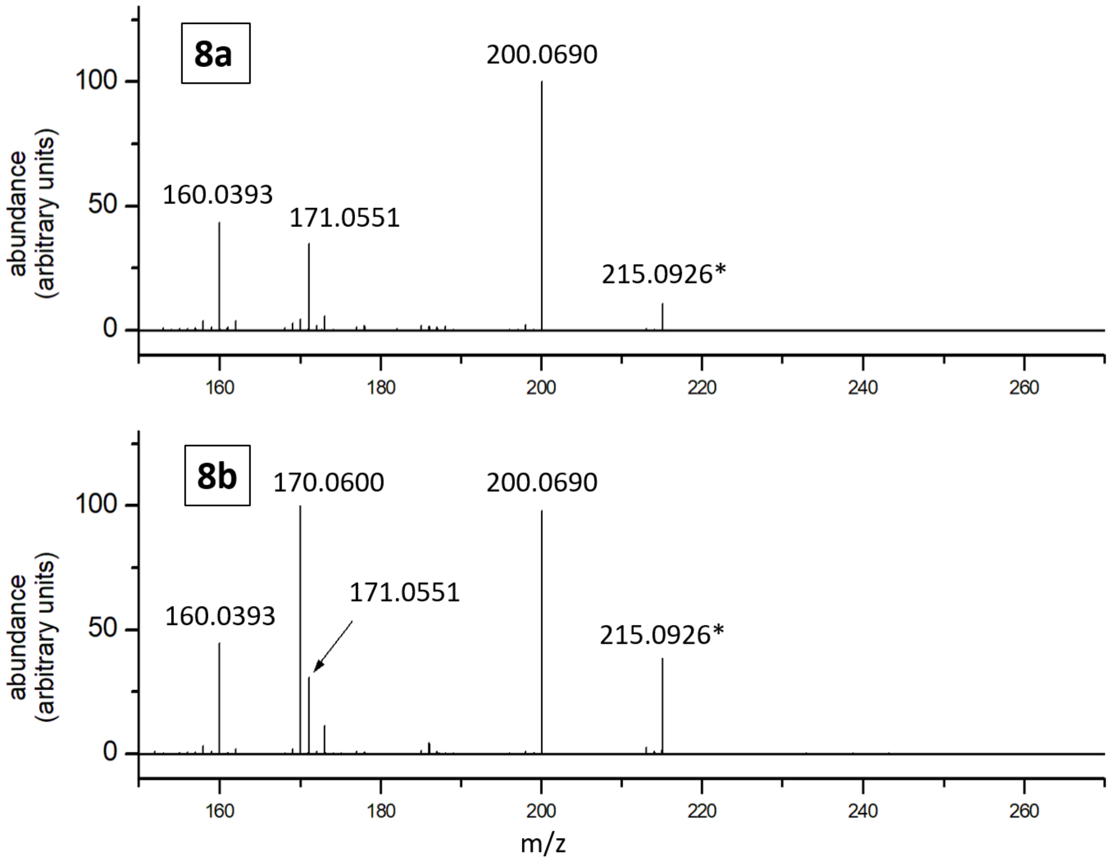

Figure S34. Fragmentation spectra of precursor ion m/z 215.0926 for isomers **8a** and **8b**. The precursor ion is marked by an asterisk. In sample **8b** the precursor ion 215.0926 evidenced an alternative fragmentation leading to the formation of fragment m/z 170.0600 which did not appear in **8a**.
